# Lipid peroxidation impacts orthoflavivirus infection in a virus-dependent manner

**DOI:** 10.1038/s41419-026-09071-8

**Published:** 2026-07-08

**Authors:** Kim Chi Thi Vu, Yvonne F. Grande, Laura Bierau, Martin Schauflinger, Emilia Spremberg, Joanne Wei Kay Ku, Julia Hehner, Anja Schöbel, Eva Herker

**Affiliations:** Institute of Virology, Marburg University, Marburg, Germany

**Keywords:** Viral infection, Cell death, Lipid peroxides

## Abstract

Orthoflavivirus infection is intricately linked to host cell lipid metabolism, yet the function of bioactive lipids as regulators of infection remains to be elucidated. Here, we investigated the role of lipid mediator pathways, namely ALOX/COX enzymes and upstream lipases, in orthoflavivirus replication by comparing dengue virus (DENV), Zika virus (ZIKV), wild-type yellow fever virus (YFV-Asibi), and the live-attenuated vaccine strain YFV-17D. DENV, ZIKV, and YFV-Asibi, but not the vaccine strain, induced COX2 expression in Huh7 hepatoma cells, correlating with prostaglandin E2 (PGE2) levels in culture supernatants. All four viruses replicated more efficiently in COX2-, ALOX15-, and MGLL-deficient cells, indicating a broadly antiviral role for these enzymes. In contrast, DENV and ZIKV specifically induced ALOX12 expression and depended on ALOX12 for efficient viral RNA replication, as demonstrated by reduced genome copy numbers, altered dsRNA replication compartment morphology, and decreased infectious titers in ALOX12-depleted cells. Direct measurement of lipid peroxidation revealed that ZIKV infection markedly elevated lipid peroxide levels through both ALOX12-dependent and -independent mechanisms, whereas DENV infection did not cause detectable lipid peroxide accumulation. Consistent with this, the ferroptosis inhibitor ferrostatin impaired DENV replication, while the ferroptosis inducer erastin enhanced it; this proviral effect of erastin was fully abolished by ALOX12 knockdown, indicating that DENV depends entirely on ALOX12-driven lipid peroxidation. Iron chelation reduced both DENV and ZIKV infection, confirming a requirement for iron-dependent oxidative processes. The proviral role of lipid peroxidation extended beyond hepatoma cells, as ferrostatin treatment significantly reduced DENV and ZIKV infection in human microglia cells. Our results reveal virus-specific exploitation of lipid peroxidation pathways by orthoflaviviruses and identify ALOX12-dependent lipid peroxidation as a novel proviral mechanism that may represent a target for antiviral intervention.

## Introduction

Orthoflaviviruses are positive-sense, single-stranded RNA viruses within the family *Flaviviridae*. Members of this genus are responsible for severe endemic and epidemic diseases worldwide, posing significant public health challenges [1]. The genus comprises more than 89 species, including several medically significant viruses such as yellow fever virus (YFV), dengue virus (DENV), Zika virus (ZIKV), West Nile virus (WNV), and Japanese encephalitis virus (JEV) [2]. These viruses are primarily transmitted by arthropod vectors, most notably *Aedes* and *Culex* mosquitoes, as well as ticks [1]. The geographic distribution of these vectors places billions of people at risk of infection [1]. Orthoflaviviral infections can be asymptomatic or may progress to severe conditions, such as hemorrhagic fever, meningoencephalitis, and other neurological complications [1]. While effective vaccines were developed for several orthoflaviviruses and have been proven highly successful in controlling YFV [3], no specific antiviral agents are available for these infections. As orthoflavivirus infections remain a significant global health burden, with potential for outbreaks in previously unaffected regions, potent antiviral therapy would be greatly beneficial in managing orthoflavivirus infections [4].

The orthoflavivirus genome consists of a single-stranded, positive-sense RNA of approximately 11 kb in length [1]. The genomic RNA contains a single open reading frame (ORF) flanked by 5′ and 3′ untranslated regions (UTRs) [5]. The ORF encodes a single polyprotein that is co- and post-translationally processed by viral and host proteases into three structural proteins, capsid (C), precursor membrane (prM), and envelope (E), and the seven nonstructural proteins NS1, NS2A, NS2B, NS3, NS4A, NS4B, and NS5 [1]. The structural proteins mediate viral attachment, entry, membrane fusion, assembly, and virion secretion, whereas the nonstructural proteins orchestrate viral RNA replication, virion morphogenesis, and evasion of innate immune defenses [1].

Orthoflavivirus replication occurs in replication organelles (ROs), which are complex invaginations of the endoplasmic reticulum (ER) membrane termed vesicle packets [6]. These viruses depend heavily on host lipid metabolism, subverting multiple cellular pathways to promote efficient RO biogenesis, viral RNA synthesis, and assembly of their enveloped virions [6–10]. We and others have previously shown that orthoflaviviruses induce profound remodeling of host cell lipids that are essential for efficient infection and replication [10–13].

Lipid mediators are bioactive molecules derived from the enzymatic oxidation of polyunsaturated fatty acids (PUFAs) that are liberated from membranes [14–16]. They can act as either pro- or anti-inflammatory agents and influence viral infections [17]. Cytosolic phospholipase A2 (cPLA2) and monoacylglycerol lipase (MGLL) are two key lipases generating free PUFAs, particularly arachidonic acid, that serve as the direct precursor for lipid mediators [14, 18]. Their biosynthesis is then driven by three enzyme families: cyclooxygenases (COX-1 and COX-2), which generate prostaglandins and thromboxanes; lipoxygenases (ALOXs), responsible for the formation of leukotrienes and hydroxyeicosatetraenoic acids (HETEs); and cytochrome P450 monooxygenases (CYPs), which produce epoxyeicosatrienoic acids (EETs) and other oxidized lipids [15, 16]. The coordinated action of these enzymes regulates the balance between pro-inflammatory and pro-resolving lipid mediators, thereby influencing the outcome of infection and tissue homeostasis [16].

In addition to their role in producing lipid mediators, ALOX enzymes can generate lipid hydroperoxides that accumulate in cellular membranes and thereby sensitize cells to ferroptosis [19, 20]. Ferroptosis is a non-apoptotic regulated form of cell death characterized by the iron-dependent accumulation of lipid peroxides within cellular membranes [21]. Central to its regulation is the Fenton reaction in which ferrous iron (Fe²⁺) catalyzes the conversion of hydrogen peroxide into highly reactive hydroxyl radicals that oxidize lipids [21]. The enzyme acyl-CoA synthetase long-chain family member 4 (ACSL4) enhances ferroptosis sensitivity by incorporating PUFAs into phospholipids, creating substrates prone to peroxidation [22]. Conversely, glutathione peroxidase 4 (GPX4) acts as a key suppressor of ferroptosis by reducing lipid hydroperoxides to non-toxic lipid alcohols, thereby maintaining membrane integrity [23]. A second parallel protective pathway against ferroptosis involves the oxidoreductase ferroptosis suppressor protein 1 (FSP1) that reduces non-mitochondrial coenzyme Q10 (CoQ10), producing a potent lipophilic antioxidant that effectively suppresses the propagation of lipid peroxides [24, 25]. Dysregulation of this balance between iron-mediated oxidative stress and GPX4/FSP1-dependent antioxidant defense ultimately determines cellular susceptibility to ferroptosis [21].

In addition to non-enzymatic lipid peroxide formation through the Fenton reaction, enzymatic peroxidation via ALOX enzymes has been linked to the induction of ferroptosis in various contexts [19, 20]. Specifically, ALOX5 is required for ferroptosis induction in neural cells affected by Huntington’s disease [26]. In cancer cells, ALOX12 expression is required for reactive oxygen species (ROS)-induced p53-mediated ferroptosis, a mechanism that is independent of GPX4 [27]. Interestingly, the pathogenic bacterium *Pseudomonas aeruginosa* can express a lipoxygenase (pLoxA) to trigger ferroptosis in human bronchial epithelial cells [28].

Previous studies have identified a role for COX2 in both viral infection-induced inflammation and the replication of viruses such as the hepatitis C virus and DENV [29, 30]. To gain more insight into lipid mediator synthesis pathways in orthoflavivirus infection, we used lentivirus-delivered small hairpin (sh)RNAs to knock down key enzymes, including ALOX/COX and lipases. All orthoflaviviruses analyzed replicated better in COX2-, ALOX15-, and MGLL- knockdown cells. Interestingly, ZIKV and DENV but not YFV strains induced ALOX12 expression and replicated less efficiently in ALOX12-deficient cells. Using ferroptosis inhibitors and inducers, we demonstrate that ALOX12 is involved in lipid peroxidation triggered by and required for DENV and ZIKV infection.

## Materials and methods

### Cell lines, culture conditions, and viability assays

Huh7 cells were provided by R. Bartenschlager, HEK293T and Vero E6 cells were obtained from the American Type Culture Collection, HMC3 from A. Slowik, THP-1 from the German Collection of Microorganisms, and BHK21 were provided by C. Munoz-Fontela. HEK293T, VeroE6, Huh7, and BHK21 cells were authenticated by STR profiling. Cells were cultivated under standard cell culture conditions in either RPMI (THP-1) or high glucose DMEM (all other cells) supplemented with 10% FCS (Gibco) and 1% GlutaMax (Gibco). Cell viability was analyzed by CellTiter96 Aqueous One Solution Reagent (Promega). Calcium phosphate precipitation was used for transfection for lentivirus production. Cell cultures were routinely tested for mycoplasma.

### Differentiation of THP-1 cells

THP-1 cells were differentiated as described [13] by interleukin 4 (IL-4) and phorbol-12-myristate-13-acetate (PMA) treatment (final concentration: IL-4 (20 ng/ml), PMA (20 ng/ml)). After 4 days, the cells were used for infection experiments with DENV and ZIKV.

### Plasmids and primers

The following plasmids were described previously: pFK-DVs encoding DENV-2 16681 [31], pJW231 and pJW232 encoding ZIKV PRVABC59 [32], pYFV-Asibi encoding YFV-Asibi [33], and pACNR-FLYFV-17Da encoding YFV-17D [34, 35]. Lentiviral shRNA constructs were cloned into pSicoR-MS1 as described previously [36, 37] using the following target sequences: ALOX5, TCAAGATCAGCAACACTATTT; ALOX12, TGGTATGCCTGGGTCCCTAAT; ALOX15, GCTATCAAAGACTCTCTAAAT; COX2, AGAGTATGCGATGTGCTTAAA; MGLL, GCACATATGTATCCATTTA; cPLA2, TCGTTTCAACCAGAACGTTAA), and shNT (target sequence GCGCGATAGCGCTAATAATT) has been described previously [38].

For the flavivirus iRFP670 reporter construct, we replaced the EGFP from the ZIKV EGFP reporter previously described [39] with iRFP670 [40] amplified by PCR using the primers iRFP fw TCGAGGTTTAAACCTGCAGGCGCGCCGCCACCATGGCGCGTAAGGTCGATCTC and iRF rev CTTACGCGTCACTTTGCGCTTTTTCTTCGGCGGGGTGCTATGCTGGCTATCCGCGGTCGCTTCATCATCGCTGCT GCGTTGGTGGTGGGCGGCGGT and SfbI and MluI for cloning into pWPI RCsec61NLSopt ZIKV Neo vector.

### Antibodies and reagents

The following antibodies were obtained commercially: anti-orthoflavivirus E protein D1-4G2-4-15 (Novus Biologicals, 1:100 IF, 1:1000 WB), GAPDH (Santa Cruz, Sc-365062, 1:500 WB), J2 dsRNA antibody (Jena Biosciences, RNT-SCI-10010200, 1:100 IF), Alexa 488-conjugated IgG (donkey, (H + L), Invitrogen, A-21208, 1:2000 IF), Hoechst 33342 (Invitrogen, H1399, 1:6000 IF), and HRP-conjugated secondary antibodies (Jackson Laboratories, 1:10,000 WB). Ferrostatin-1 (10 mM stock in DMSO, f.c. 40 µM) and erastin (10 mM stock in DMSO, f.c. 1 µM) were obtained from MedChem, deferoxamine-mesylate (DFX) (252750-1GM, 100 µM) and tert-butyric acid (30 M, f.c. 50 µM) from Sigma-Aldrich, 12-(S)-HETE (297 µM stock in ethanol, f.c 10 nM), 15-(S)-HETE (297 µM stock in ethanol, f.c 10 nM), and the PGE2 ELISA (Cay500141-96) from Cayman. Enzymes for molecular cloning were purchased from New England Biolabs.

### In vitro transcription of orthoflavivirus RNA, production of virus stocks, and infection

For the preparation of DENV and YFV stocks, the corresponding plasmids were linearized by restriction enzyme digestion and purified by phenol-chloroform extraction. ZIKV stocks were generated using a two-plasmid system combined with an overlap PCR approach using the primers ZIKV pJW231 BamHI fw TAGGATCCTAATACGACTCACTATAG; ZIKV Conserved 3499 Rev, GCCTTATCTCCATTCCATACCA; ZIKV pJW232 ApaLI fw AGGGAGTGCACAATGCCCCCA; ZIKV pJW232 EcoRI rev CTAGAATTCGCCCTTGCTCCG as described [13]. The resulting overlap PCR products were purified by gel extraction. In vitro transcription was carried out using the MEGAscript SP6 Transcription Kit with Cap Analog (m⁷G(5′)ppp(5′)G) (Thermo Fisher Scientific) for DENV and YFV-17D, and the HiScribe T7 ARCA mRNA Kit (New England Biolabs) for ZIKV and YFV-Asibi. P0 stocks were produced in Vero E6 cells. Briefly, 4 × 10⁶ cells were washed with Opti-MEM (Life Technologies) and resuspended in 400 µl of cytomix buffer (120 mM KCl, 5 mM MgCl_2_, 0.15 mM CaCl_2_, 2 mM EGTA, 1.9 mM ATP, 4.7 mM GSH, 25 mM HEPES, 10 mM potassium phosphate buffer, pH 7.6). The cell suspension was mixed with 10 µg of in vitro–transcribed orthoflavivirus RNA and electroporated at 260 V and 950 µF using a Gene Pulser II (Bio-Rad). To generate P1 virus stocks, naïve Vero E6 cells were infected with P0 virus supernatants. For infection, virus stocks were diluted either in RPMI supplemented with 10% FCS (THP-1) or in DMEM supplemented with 3% FCS (Huh7) or 10% FCS (HMC3) and added to the cells for 3 h (THP-1) or 1 h (all other cell lines) at 37 °C.

### Determination of viral titers (TCID_50_)

BHK21 cells were seeded at a density of 6 × 10^3^ cells/well in a 96-well plate. One day post-seeding, the cells were infected with serially diluted culture supernatants for which the viral titers were to be determined. Uninfected BHK21 cells served as a control. After 5 days, cells were fixed with 4% PFA for 1 h, plates were stained with crystal violet, and the TCID_50_ was calculated using the Reed and Muench calculator [41].

### Lentivirus production and transduction

Lentiviral particles were generated in 293 T cells by co-transfection of pSicoR-MS1 shRNA constructs or the flavivirus iRFP reporter construct with the helper plasmid pCMVΔR8.91 and pMD.G expressing the glycoprotein of vesicular stomatitis virus [36]. Lentiviral pseudoparticles were concentrated by ultracentrifugation (Beckman-Coulter). Transduction was performed in medium supplemented with 4 μg/ml polybrene, and stocks were titrated on Huh7 cells using flow cytometry (Guava easyCyte HT Cytometer) or microscopy.

### CPE assay

Huh7 cells were transduced with lentiviral particles expressing the different shRNAs. Four days post-transduction (dpt), cells were infected with orthoflavivirus stocks. Three days post-infection (dpi), cells were fixed, stained with crystal violet to assess the cytopathic effect (CPE), imaged on a ChemiDoc (Bio-Rad), and quantified using Fiji.

### Immunoblot analysis

Cells were lysed on ice for 1 h in RIPA buffer (150 mM NaCl, 50 mM Tris-HCl, pH 7.6, 1% NP-40, 0.5% sodium deoxycholate, 0.1% SDS, 1 mM EDTA, protease inhibitor cocktail (Sigma), 1 mM phenylmethylsulfonyl fluoride (PMSF) (AppliChem)). Lysates were separated by SDS-PAGE, transferred to nitrocellulose membranes (Amersham Protran, Cytiva), and probed with antibodies. Proteins were detected by chemiluminescence using Immobilon Classico or Immobilon Forte (Merck), Lumi-Light substrate (Roche), or SuperSignal West Femto (Thermo Fisher) on a ChemiDoc system (Bio-Rad), and band intensities were quantified with Image Lab/Fiji. Full-length immunoblots are available in the Supplementary Material.

### RNA isolation and quantitative RT-PCR

Total RNA was isolated using TriReagent (Sigma-Aldrich) and treated with rDNase I (TURBO DNA-free kit, Ambion) to remove genomic DNA. Viral RNA (vRNA) from the culture supernatant was isolated using NucleoSpin RNA Virus Kit (Machery Nagel) according to the manufacturer’s protocol. cDNA was synthesized with random hexamers (Qiagen), SuperScript III reverse transcriptase, and RNaseOut (Thermo Fisher). qPCR was performed using Luna Universal qPCR Master Mix (New England Biolabs) on a StepOnePlus Real-Time PCR system (Applied Biosystems) using the following primer sets [42]: 18S rRNA fw, GTAACCCGTTGAACCCCATT; 18S rRNA rev, CCATCCAATCGGTAGTAGCG; ALOX5 fw, CTCAAGCAACACCGACGTAAA; ALOX5 rev, CCTTGTGGCATTTGGCATCG; ALOX12 fw, AGTTCCTCAATGGTGCCAAC; ALOX12 rev, GCAGCCAGGTATTGCTTCTC; ALOX15 fw, GGGCAAGGAGACAGAACTCAA; ALOX15 rev, CAGCGGTAACAAGGGAACCT; COX2 fw, GTTCCACCCGCAGTACAGAA; COX2 rev, AGGGCTTCAGCATAAAGCGT; MGLL fw, AATGCAGACGGACAGTACCTC; MGLL rev, GAGCCAGCTCTTCATAGCGG; cPLA2 fw, ATGGATGAAACTCTAGGGACAGC; cPLA2 rev, CTGGGCATGAGCAAACTTCAA (all from Harvard Primer Bank) [42]; ZIKV fw, TTCGGAATATGGAGGCTGAG; ZIKV rev, TCGTTTGAGCCTATCCCATC; DENV fw, GCAGAAACACAACATGGAACRATAGT; and DENV rev, TGATGTAGCTGTCTCCRAATGG.

### Immunofluorescence microscopy

For immunofluorescence microscopy, cells seeded on coverslips were infected and fixed at 48 hpi in 4% paraformaldehyde (PFA). After permeabilization for 6 min with 0.1% Triton X-100 in PBS and incubation in blocking solution (5% BSA, 1% fish skin gelatin, 50 mM Tris in PBS), cells were stained overnight with a dsRNA antibody, followed by an Alexa Fluor-conjugated secondary antibody. Nuclei were stained with Hoechst, and coverslips were embedded in Mowiol mounting media. Images were acquired on a Leica Stellaris 8 confocal microscope equipped with an HC PL APO 63x objective (1.40 NA, oil) and a white light laser. dsRNA foci were quantified using the particle analyzer function of Fiji.

### Live cell imaging

Liperfluo microscopy of DENV-, ZIKV-, and YFV-infected cells: Huh7 cells were transduced with the lentiviral expression system for the orthoflavivirus iRFP reporter construct for 4 days prior to infection with orthoflavivirus stocks. At 48 hpi, fresh Leibovitz’s medium containing 5 µM Liperfluo was added to the cells. Cells were incubated for 1 h at 37 °C with 5% CO_2_ prior to visualization by microscopy. Lipid peroxidation levels were assessed using Liperfluo green fluorescence, representing the oxidized form, while iRFP fluorescence from the orthoflavivirus reporter was used to distinguish infected and uninfected cells. The experiment was performed using a Nikon Ti2-E widefield microscope equipped with a Nikon CFI Plan Apo 100x Lambda Objective, pE4000 LEDs, Quad LED ET filter sets, and a DS-Qi2 camera. Liperfluo microscopy of ZIKV-infected cells expressing shRNAs: Huh7 cells expressing the orthoflavivirus iRFP reporter were transduced with lentiviruses expressing shALOX12 or shNT in combination with mCherry 4 days prior to infection with ZIKV stocks. At 48 hpi, fresh Leibovitz’s medium containing 5 µM Liperfluo was added to the cells and incubated for 30 min at 37 °C with 5% CO_2_. Only cells expressing the shRNA marker mCherry and the orthoflavivirus iRFP reporter were used for quantification. Images were acquired on a Leica Thunder Live Cell Imager equipped with a HC PL APO 63x/1.20 W CORR objective, LED8, Quad LED filter sets, and a K8 camera. Corrected total cell fluorescence (CTCF) of oxidized Liperfluo was determined using Fiji. For relative quantification, infected and uninfected cells within the same field of view were analyzed.

### Electron microscopy

For electron microscopy, cells were grown on sapphire discs (Engineering Office M. Wohlwend GmbH, Sennwald, Switzerland). The cells were fixed for 1 h in 0.1 M Na-cacodylate buffer pH 7.2 containing EM-grade 2.5% glutaraldehyde and 2% formaldehyde, followed by washing and incubation in 1% osmium tetroxide and 1.5% ferricyanide for 1 h in the same buffer. The cells were incubated in 2% uranyl acetate in water overnight, dehydrated through an ethanol series into acetone, and subsequently infiltrated with resin (EMbed-812 Kit with BDMA), and polymerized at 60 °C for 48 h. The sapphire disc was removed to expose the cells at the blockface. Sections with a nominal thickness of 70 nm were cut using a Leica EM UC6 ultra- microtome and an ultra-45 ° diamond knife (Diatome), and mounted on formvar-coated single slot nickel grids. Sections were subsequently stained with 2% uranyl acetate followed by 3% lead citrate. Samples were imaged at an acceleration voltage of 120 kV in a JEOL JEM-1400 TEM equipped with a TemCam-F416 camera (TVIPS, Gauting, Germany; unless otherwise mentioned, the materials are from Science Services, Munich, Germany).

### Quantification and statistical analysis

No sample size calculation was performed. In general, a minimum of three independent experiments was performed to enable statistical analysis. Sample sizes (n) represent independent experiments if not stated otherwise. The exact sample sizes are stated in the figure legends. Data were only excluded when the quality of the samples was not sufficient (i.e., deviating house-keeping gene expression); for immunofluorescence image analysis, only cells with an intact nucleus were used for quantification. The investigators were not blinded in this study. Blinding was not possible as the experiments were performed by individual investigators who were aware of the experimental groups. R [43] and RStudio [44] were used to visualize data and for statistical analysis. Statistical analysis was performed using unpaired two-tailed Student’s *t*-test, unpaired two-tailed Welch’s unequal variances *t*-test, unpaired two-tailed Mann–Whitney *U* test, or, in case of normalized data, two-tailed one-sample Student’s *t*-test as indicated in the figure legends. Data were analyzed and visualized using several packages available for RStudio, namely gdata (version 3.0.1), dplyr (version 1.1.4) [45], tidyr (version 1.3.1) [46], ggplot2 (version 4.0.0) [47], coin (version 1.4-3) [48], ggsignif (version 0.6.4) [49], and ggbeeswarm (version 0.7.2) [50].

## Results

### ALOX12 expression is induced in DENV and ZIKV but not YFV infection

**Fig. 1:**
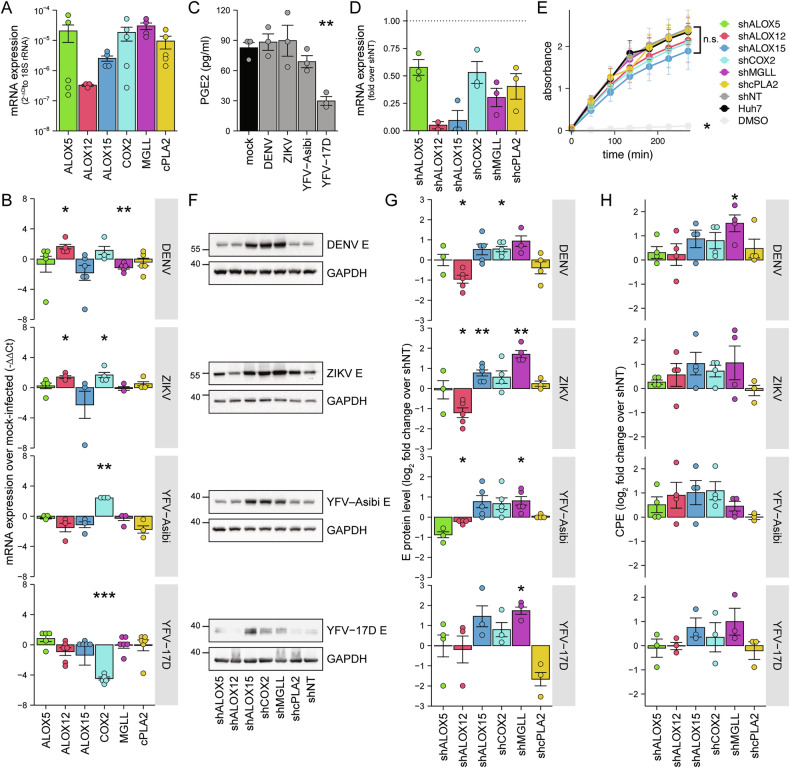
Disruption of the ALOX/COX pathway impacts orthoflavivirus infection. **A** Quantitative RT-PCR analysis of *ALOX5*, *ALOX12*, *ALOX15*, *COX2*, *MGLL*, and *cPLA2* mRNA levels in Huh7 cells. Data are normalized to *18S rRNA* expression (Mean ± SEM, *n* = 4–5). **B** Huh7 cells were infected with orthoflavivirus stocks (MOIs DENV: 0.05, ZIKV: 0.1, YFV-Asibi: 0.2, YFV-17D: 0.005) and at 48 h post-infection (hpi), total RNA was isolated and mRNA expression level of target proteins determined using RT-qPCR (Mean ± SEM, *n* = 3–6, *p ≤ 0.05, **p ≤ 0.01, two-tailed one-sample Student’s *t*-test). **C** PGE2 levels in supernatants of mock- or orthoflavivirus-infected Huh7 cells (MOIs DENV: 0.02, ZIKV: 0.03, YFV-Asibi: 0.015, YFV-17D: 0.002) at 72 hpi were measured by ELISA (Mean ± SEM, *n* = 3; **p ≤ 0.01, unpaired two-tailed Welch’s *t*-test). **D** Huh7 cells were transduced with shRNAs and knockdown efficiency was determined by RT-qPCR (Mean ± SEM, *n* = 3). **E** Determination of cell viability of shRNA-transduced knockdown cells at 5 dpt using MTT assay (Mean ± SD, *n* = 3, *p* ≤ 0.05, two-way ANOVA followed by BH-corrected post-hoc contrasts vs. shNT). **F** Representative immunoblots of viral E protein levels in Huh7 cells infected with DENV, ZIKV, YFV-Asibi, or YFV-17D (MOIs DENV: 0.02, ZIKV: 0.03, YFV-Asibi: 0.015, YFV-17D: 0.002) and lysed at 72 hpi. GAPDH served as a loading control. **G** Densitometric quantification of E protein level. Shown is the signal intensity normalized to GAPDH as log_2_ fold change over shNT (Mean ± SEM, *n* = 3–5; *p ≤ 0.05, **p ≤ 0.01, two-tailed one-sample Student’s *t*-test). **H** shRNA-transduced Huh7 cells were infected with orthoflavivirus stocks (MOIs DENV: 0.02, ZIKV: 0.03, YFV-Asibi: 0.015, YFV-17D: 0.002). Cells were fixed at 3 dpi and stained with crystal violet to visualize cytopathic effects (CPE). Calculation of CPE was performed by quantifying the mean grey intensity using Fiji. Plot indicates CPE as log_2_ fold change over shNT (Mean ± SEM, *n* = 3–4. *p < 0.05, two-tailed one-sample Student’s *t*-test).

To investigate the lipid mediator synthesis pathways in flavivirus infection, we focused on the key enzymes, including ALOX/COX (ALOX5/12/15 and COX2) and lipases (MGLL and cPLA2), as they are involved in both pro- and anti-inflammatory lipid mediator synthesis in cells [14–16, 18]. We used the Huh7 hepatoma cell line for our experiments, as these cells are equally permissive for all human-pathogenic orthoflaviviruses we planned to study and show a high lentiviral transduction efficacy suitable for screening. First, to determine the expression of the selected enzymes in Huh7 cells, mRNA expression levels of target proteins were quantified by RT-qPCR. All enzymes were expressed in Huh7 cells, albeit at different levels (Fig. 1A). Next, Huh7 cells were infected with DENV, ZIKV, and two YFV strains, the Asibi wildtype strain and the live attenuated cell culture–adapted vaccine strain YFV-17D. Interestingly, ALOX12 mRNA, which was expressed only at very low levels in naïve cells, significantly increased during DENV and ZIKV infection (approximately 2.5-fold increase for both viruses), while no changes were observed for both YFV strains (Fig. 1B). This suggests a more prominent role for ALOX12 in replication of DENV and ZIKV compared to YFV.

### YFV-17D vaccine strain reduces COX2 expression and PGE2 production

We additionally observed a striking difference between the YFV vaccine strain and wild-type orthoflaviviruses: Whereas infection with DENV, ZIKV, and YFV-Asibi led to an increase in COX2 levels, YFV-17D-infected cells had significantly lower COX2 expression compared to uninfected cells (Fig. 1B). COX2 mediates the conversion of arachidonic acid (AA) to prostaglandin H2 (PGH2), the precursor of various prostaglandins [15]. Thus, we examined whether COX2 expression correlated with prostaglandin production and measured the levels of secreted PGE2. YFV-17D infection led to a significant decrease in PGE2 secretion, while infection with the other viruses exhibited no changes in PGE2 levels (Fig. 1C). This correlates with the lower COX2 mRNA levels during YFV-17D infection (Fig. 1B). However, although YFV-Asibi, DENV, and ZIKV upregulated COX2 expression (Fig. 1B), we did not observe a change in the levels of PGE2 released compared to the uninfected control (Fig. 1C), suggesting that additional regulatory mechanisms may be involved in PGE2 production during orthoflavivirus infection. Notably, YFV-17D infection uniquely suppresses COX2 expression and PGE2 release.

### ALOX15/COX/MGLL enzymes limit orthoflavivirus infection, while ALOX12 is required for efficient DENV and ZIKV infection

In order to investigate whether the enzymes involved in lipid mediator production affect orthoflavivirus infection in Huh7 cells, we used lentiviral vectors expressing shRNAs to deplete the respective target enzymes. Prior to infection experiments, we validated knockdown efficiencies via RT-qPCR at 5 dpt. The reduction in mRNA expression was over 90% for ALOX12 and ALOX15 and between 50–70% for ALOX5, COX2, MGLL, and cPLA2 (Fig. 1D). Using MTT assays, we observed that depletion of ALOX15 slightly reduced cell viability compared to the controls while all other shRNAs did not affect cell viability (Fig. 1E). Next, we infected Huh7 cells transduced with the different shRNA-expressing lentiviral vectors with DENV, ZIKV, and the two YFV strains, and analyzed virus replication by immunoblotting as well as by determining the cytopathic effects (CPE) of infection. ALOX15-, COX2-, and MGLL-depleted cells displayed elevated viral E protein levels compared to shNT controls at 48 hpi for all orthoflaviviruses analyzed (Fig. 1F, G). In shALOX5- and shcPLA2-expressing cells, E protein levels were comparable to the shNT control, except for reduced YFV-17D E protein levels in cPLA2-depleted cells. Interestingly, ALOX12 knockdown led to a two-fold decrease in E protein levels during DENV and ZIKV infection, whereas only a marginal decrease was observed in YFV-Asibi-infected cells (Fig. 1F, G). Next, we performed CPE assays as an alternative method to assess viral replication. CPE is, however, a late-stage event that reflects cumulative cell death, whereas E protein levels at 48 hpi provide a more direct and early readout of viral replication. In line with increased E protein levels detected by immunoblot analysis, a slight increase in CPE was observed in crystal violet staining of ALOX15-, COX2-, and MGLL-knockdown cells, while the knockdown of ALOX5 and cPLA2 showed no changes compared to the control (Figs. 1H and S1). For ALOX12-knockdown cells, there was a slight increase in CPE during YFV-Asibi and ZIKV infection, while no differences were observed for DENV and YFV-17D (Fig. 1H). Of note, the experimental setup was more likely to detect an increase in CPE as the CPE in shNT-control cells was barely detectable when almost all cells were already dead in shCOX2-expressing cells. We additionally collected the supernatants 48 hpi and determined extracellular infectious titers using TCID_50_ assays. However, due to high variability between samples, no significant changes in extracellular orthoflavivirus titers were detected (Fig. S2). This variability in TCID_50_ data (Fig. S2) likely reflects the biological variation inherent to testing multiple shRNA and virus combinations, as well as the high coefficient of variation of endpoint dilution assays.

Taken together, our findings indicate that ALOX15, COX2, and MGLL exert modest antiviral effects in orthoflavivirus-infected Huh7 cells. They also suggest that ALOX12 may play a role in the replication of DENV and ZIKV but not of YFV.

### The main lipid mediator products of ALOX12 and ALOX15 do not significantly affect orthoflavivirus replication

As ALOX12 and ALOX15 had opposing effects on DENV and ZIKV replication, we wondered whether the main bioactive lipids produced by these enzymes, 12(S)-HpETE and 15(S)-HpETE, would have the opposite effect on virus infection. We therefore treated DENV- and ZIKV-infected Huh7 cells with 12(S)-HpETE and 15(S)-HpETE and analyzed virus infection by immunoblotting and TCID_50_ titration. Neither treatment significantly affected DENV or ZIKV infection (Fig. S3).

### ALOX12 is required for efficient ZIKV and DENV RNA replication

**Fig. 2:**
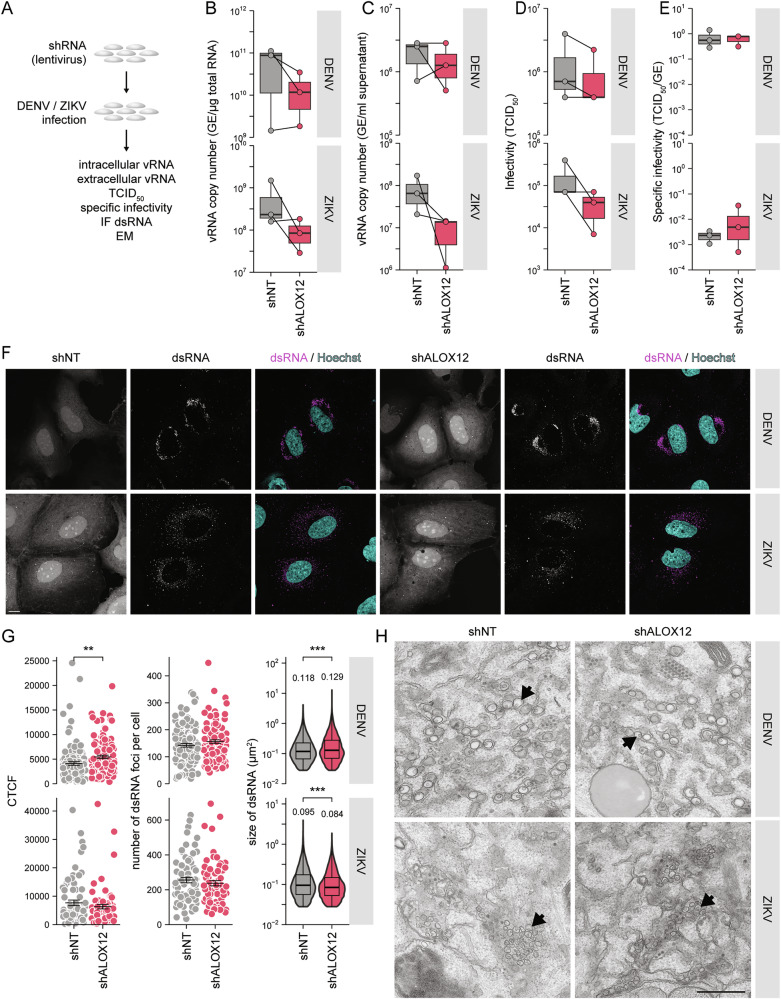
Knockdown of ALOX12 likely impairs ZIKV and DENV RNA replication. **A** Scheme of the experiments: Huh7 were transduced with lentiviruses encoding for non-targeting control shRNA (shNT) or shRNA against *ALOX12* (shALOX12), infected with DENV or ZIKV (MOIs DENV: 0.05, ZIKV: 0.1), and analyzed for viral replication. Total RNA was isolated at 48 hpi, and intracellular vRNA (**B**) as well as vRNA in the supernatant (**C**) were determined by qRT-PCR. Shown are genome equivalents (GE)/μg total RNA or GE/ml supernatant. **D** Virus titers were determined by TCID_50_ titration at 48 hpi. **E** Calculation of ZIKV and DENV specific infectivity (ratio of TCID_50_ to vRNA copy number) of shALOX12 or shNT cells at 48 hpi. Box plots indicate median (center line), upper and lower quartiles (box limits), interquartile range (whiskers) (*n* = 3, vRNA: unpaired two-tailed Welch´s *t*-test, TCID_50_ and specific infectivity: unpaired two-tailed Mann–Whitney *U* test). **F**, **G** Huh7 cells expressing shNT or shALOX12 were infected with DENV or ZIKV (MOIs DENV: 1, ZIKV: 1). Cells were fixed 48 hpi, stained with antibodies against double-stranded RNA (dsRNA) and Hoechst to visualize nuclei, and analyzed by confocal microscopy. Shown are representative images (**F**, scale bar 10 μm) and quantification of dsRNA signal as corrected total cell fluorescence (CTCF) and dsRNA foci number and size using the particle analyzer function of Fiji (23–42 cells per condition from three independent experiments, CTCF and number of dsRNA foci: mean ± SEM, **p ≤ 0.01, unpaired two-tailed Student’s *t*-test, dsRNA foci size: box plots indicate median (center line), upper and lower quartiles (box limits), interquartile range (whiskers), ***p ≤ 0.001, unpaired two-tailed Mann–Whitney *U* test). **H** shRNA-expressing infected cells (MOIs DENV: 1, ZIKV: 2) were fixed 48 hpi and processed for EM. Shown are representative images (scale bar = 500 nm). Arrows indicate vesicle packets.

In order to gain more insight into the function of ALOX12 in DENV and ZIKV replication, we next isolated total RNA and compared intra- and extracellular viral RNA (vRNA) levels, extracellular infectivity, as well as the specific infectivity of DENV and ZIKV particles (Fig. 2A). Knockdown of ALOX12 slightly reduced viral genome copy numbers within the cells (Fig. 2B), which correlates well with the reduced viral E protein levels (Fig. 1G). vRNA levels were reduced to a similar extent in the supernatant of ALOX12-deficient cells compared to controls (Fig. 2C). Likewise, viral titers were slightly reduced in shALOX12-compared to shNT-expressing cells (Fig. 2D). Consequently, the specific infectivity of released virions was unaltered (Fig. 2E). As viral RNA replication is therefore likely impaired in ALOX12-deficient cells, we next analyzed on a subcellular level viral RNA replication by staining with antibodies that detect dsRNA. These antibodies recognize dsRNA that is formed during RNA virus replication and present as a very distinct staining pattern for different viruses. In the case of ZIKV and DENV, dsRNA staining patterns differ as they are clustered in the perinuclear region in DENV-infected cells and more spread out through the cells in ZIKV-infected cells (Fig. 2F). To compare ALOX12-deficient cells to the controls, we quantified the dsRNA signal by determining CTCF as well as the number of dsRNA foci per cell and the foci size. For DENV, we detected an aberrant dsRNA signal with significantly higher CTCF values and larger dsRNA foci in ALOX12-deficient cells compared to control (Fig. 2G). In contrast, ZIKV-infected dsRNA foci were significantly smaller in shALOX12-expressing vs control cells (Fig. 2G). To evaluate if vesicle packets of the ROs have an abnormal morphology, we also performed electron microscopy studies. Here, we observed that on an ultrastructural level, the vesicle packets of both DENV and ZIKV did not appear different between ALOX12-deficient and control cells (Fig. 2H).

These results suggest that ALOX12 deficiency influences DENV RNA replication organelles without substantially altering vesicle packet structure.

### ZIKV infection causes a striking accumulation of lipid peroxides

**Fig. 3:**
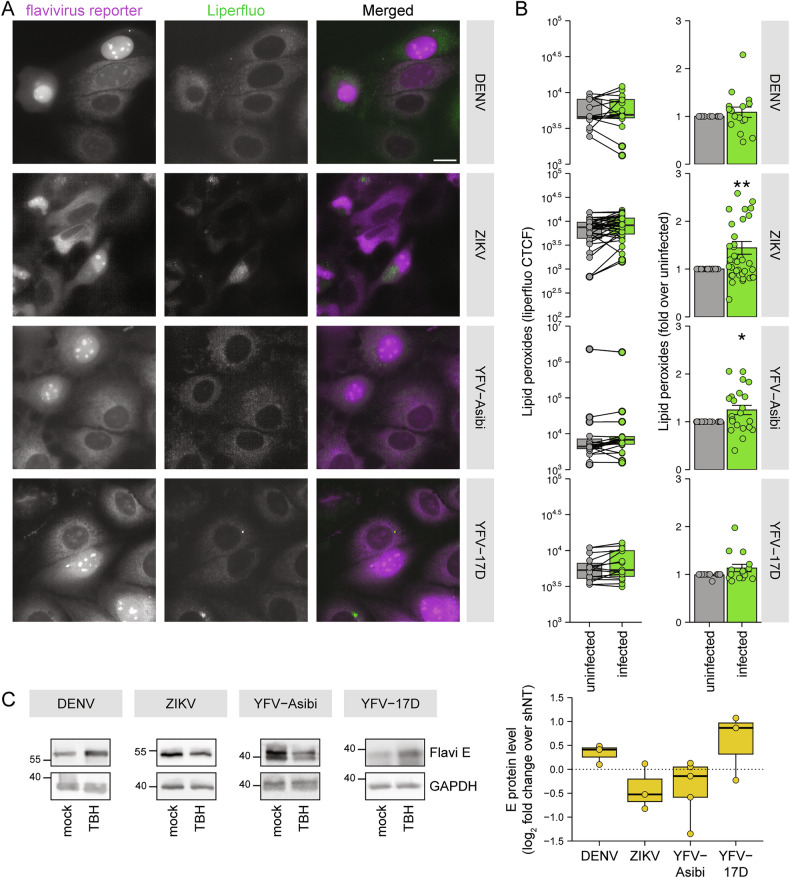
Lipid peroxides accumulate in ZIKV- and YFV-infected Huh7 cells. **A** Huh7 cell line expressing an iRFP-based infection reporter was infected with orthoflavivirus stocks (MOIs DENV: 0.05, ZIKV: 0.1, YFV-Asibi: 0.02, YFV-17D: 0.005). At 48 hpi, Leibovitz’s medium containing 5 µM Liperfluo was added to the cells, and after 1 h incubation, the cells were analyzed by wide-field microscopy. Green fluorescence represents the oxidized form of Liperfluo, while nuclear iRFP localization indicates infection (scale bar 5 µm). **B** Quantification of lipid peroxide levels as corrected total cell fluorescence (CTCF). For the raw data, each line represents paired measurements in one image. Box plots indicate median (center line), upper and lower quartiles (box limits), and interquartile range (whiskers). CTCF values of infected cells were normalized to uninfected cells within the same field of view (Mean ± SEM, 11–38 cells from three independent experiments, **p ≤ 0.01, two-tailed one-sample Student’s *t*-test). **C** Immunoblot analysis of viral E protein expression in control-treated or tert-butyl hydroperoxide (TBH)-treated Huh7 cells infected with DENV, ZIKV, YFV-Asibi, or YFV-17D for 48 h (MOIs DENV: 0.02, ZIKV: 0.03, YFV-Asibi: 0.2, YFV-17D: 0.005). GAPDH served as a loading control. Quantification of viral E protein levels (log_2_ fold change over mock-treated samples) after TBH treatment (box plots indicate median (center line), upper and lower quartiles (box limits), and interquartile range (whiskers), *n* = 3).

Given that ALOX12 expression was required for efficient DENV and ZIKV replication but ALOX12-derived lipid mediators had no effect, we wondered whether this effect was due to a function of ALOX12 that is unrelated to lipid mediator synthesis, namely its involvement in the oxidation of PUFAs during ferroptosis. To investigate if orthoflavivirus infection increases lipid peroxidation levels, live cell imaging was performed using the Liperfluo sensor. Liperfluo is a non-fluorescent lipophilic sensor that fluoresces after oxidation [51]. To distinguish infected from uninfected neighboring cells under the same culture conditions, we used a cell-based orthoflavivirus reporter. This reporter consists of a fluorescent protein (iRFP) fused to an NLS and followed by a linker containing an orthoflaviviral protease cleavage site and an ER-transmembrane domain. In naïve cells, the reporter remains anchored in the ER, but upon infection, viral protease cleavage releases the NLS-iRFP, enabling clear identification of infected and uninfected cells within the same field of view. We determined CTCF of the Liperfluo channel in infected cells (iRFP signal in the nucleus) and compared it to neighboring uninfected cells (reticular iRFP signal). ZIKV- and YFV-infected Huh7 cells displayed an increase in lipid peroxide levels (Fig. 3A, B). When normalized to uninfected cells, ZIKV- and YFV-Asibi-infected cells showed a significant increase in lipid peroxides, with ZIKV having an even more pronounced effect (Fig. 3B). No differences in lipid peroxide levels were observed between DENV- and YFV-17D-infected compared to uninfected cells. Next, in order to further investigate the role of lipid peroxidation in orthoflavivirus infection, infected cells were treated with tert-butyric acid (TBH), an inducer of intracellular ROS, which in turn can promote lipid peroxidation. For DENV and YFV-17D, the levels of E protein increased during TBH treatment, whereas YFV-Asibi and ZIKV infection showed a slight reduction (Fig. 3C).

Therefore, DENV and YFV-17D benefit from elevated ROS levels while ZIKV and YFV-Asibi, which both induce lipid peroxidation themselves, do not.

### Ferroptosis inhibitors reduce DENV and YFV infection, while inducers fuel DENV and ZIKV infection

**Fig. 4:**
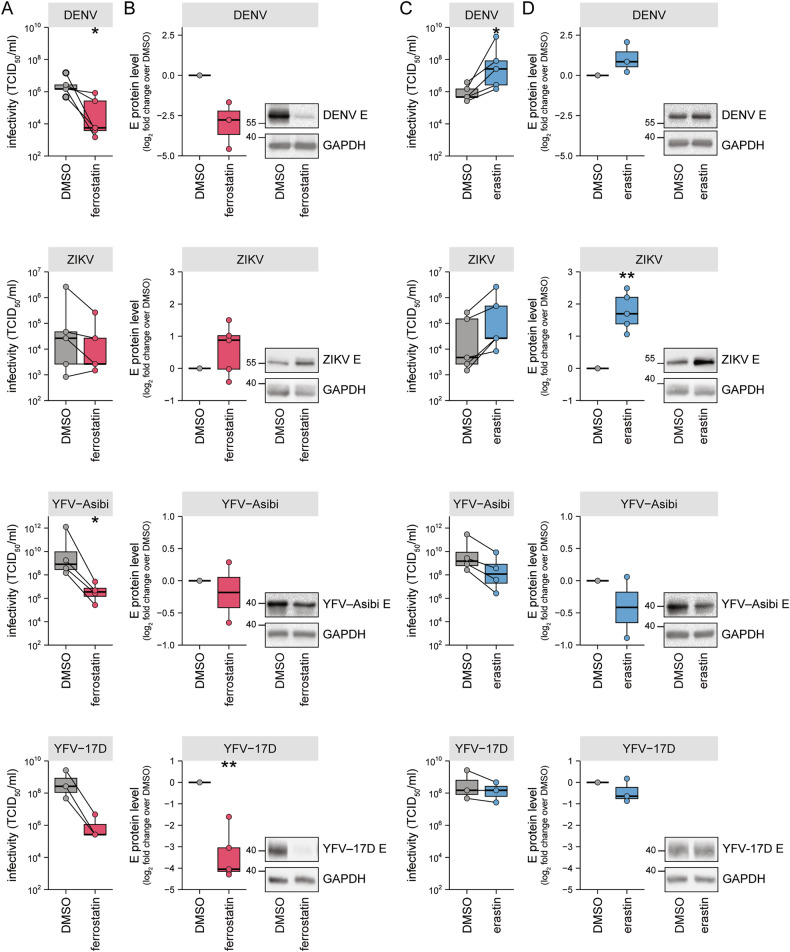
Ferroptosis inhibitor or inducer treatment affects orthoflavivirus infection. Huh7 cells were infected with orthoflavivirus stocks (MOIs DENV: 0.02, ZIKV: 0.03, YFV-Asibi: 0.015, YFV-17D: 0.002) and treated with the ferroptosis inhibitor ferrostatin (**A**, **B**) or the ferroptosis inducer erastin (**C**, **D**). Viral titers were determined at 72 hpi by TCID_50_ titration (**A**, **C**) and viral E protein levels were assessed at 48 hpi by immunoblot analysis followed by densiometric quantification (log_2_ fold change over DMSO-treated controls). GAPDH was used as loading control (**B**, **D**). Box plots indicate median (center line), upper and lower quartiles (box limits), and interquartile range (whiskers) (*n* = 3–5, *p ≤ 0.05, **p ≤ 0.01, TCID_50_: unpaired two-tailed Mann–Whitney *U* test, immunoblot: two-tailed one-sample Student’s *t*-test).

To gain more insight into the role of lipid peroxidation in orthoflavivirus infection, we treated orthoflavivirus-infected Huh7 cells with ferrostatin (a ferroptosis inhibitor) or erastin (a ferroptosis inducer) and determined viral replication by TCID_50_ titration and immunoblotting of cell lysates. In DENV-infected Huh7 cells, treatment with ferrostatin led to decreased viral titers and viral E protein levels compared to the control cells ( > 2 log TCID_50_/ml reduction) (Fig. 4A, B), while an increase was observed with the lipid peroxide inducer (Fig. 4C, D). This suggests that, similar to ROS, lipid peroxides promote DENV infection. Notably, the reduction was considerably more pronounced with ferrostatin treatment than with ALOX12 deficiency.

In contrast, no changes in infectivity and only a slight increase in E protein levels were observed with ferrostatin treatment during ZIKV infection (Fig. 4A, B). It is possible that ferrostatin treatment was not sufficient to inhibit viral replication, as ZIKV-infected cells have significantly higher levels of lipid peroxides (Fig. 3B). Conversely, treatment with erastin led to an increase in ZIKV replication (Fig. 4C, D). For YFV, treatment with the lipid peroxide scavenger led to a pronounced reduction in YFV-Asibi and YFV-17D infectious viral titers and E protein levels (Fig. 4). Interestingly, and in contrast to DENV and ZIKV, treatment with erastin also slightly decreased YFV infection (Fig. 4). These results suggest that the levels of lipid peroxides in cells are critical for orthoflavivirus infection, but the requirements differ between the viruses.

### ALOX12 is involved in lipid peroxidation triggered by and needed for DENV and ZIKV infection

**Fig. 5:**
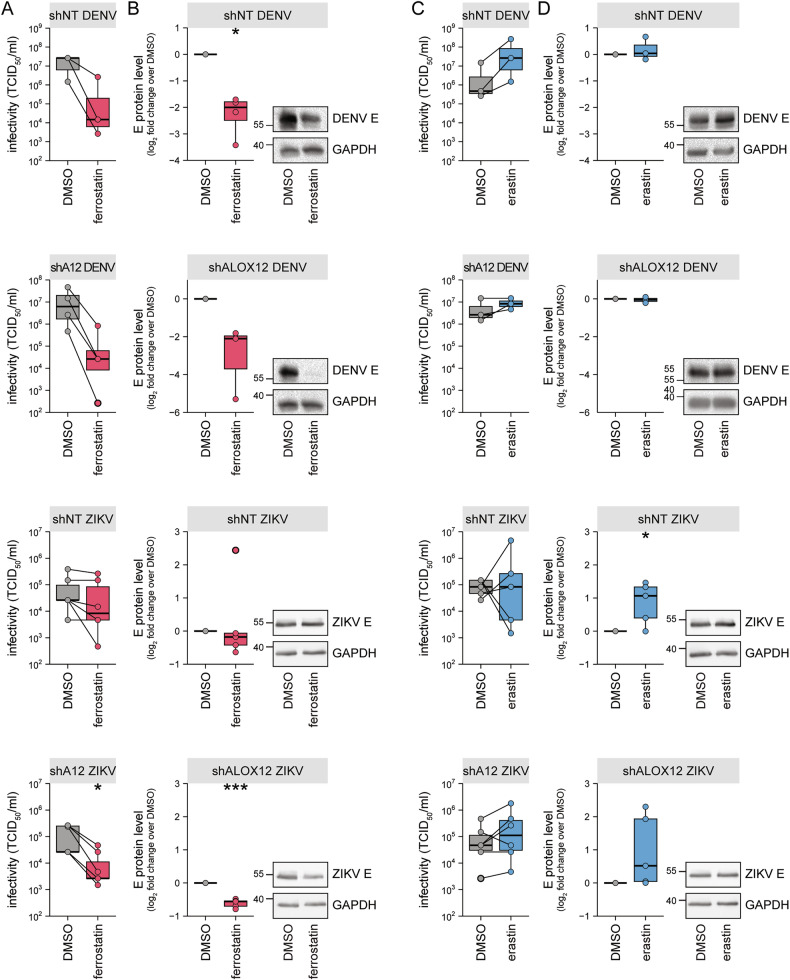
DENV and ZIKV differ in their requirement for ALOX12-generated lipid peroxides. Huh7 cells expressing non-targeting control shRNA (shNT) or shRNA against *ALOX12* (shALOX12) were infected with DENV or ZIKV (MOIs DENV: 0.02, ZIKV: 0.03). Infected Huh7 cells were treated with ferrostatin (**A**, **B**) or erastin (**C**, **D**). Viral titers were determined at 72 hpi by TCID_50_ titration (**A**, **C**), and viral E protein levels were assessed at 48 hpi by immunoblot analysis followed by densiometric quantification (log_2_ fold change over DMSO-treated controls). GAPDH was used as a loading control (**B**, **D**). Box plots indicate median (center line), upper and lower quartiles (box limits), interquartile range (whiskers), and outliers (points) (*n* = 3–5, *p ≤ 0.05, **p ≤ 0.01, TCID_50_: unpaired two-tailed Mann–Whitney *U* test, immunoblot: unpaired two-tailed Student’s *t*-test).

In DENV- and ZIKV-infected Huh7 cells, we observed upregulation of ALOX12 expression and knockdown of ALOX12 resulted in reduced replication for both viruses (Fig. 1B, G). These findings highlight the potential importance of ALOX12 in DENV and ZIKV infection. To further investigate the interplay of ALOX12 with lipid peroxidation inhibitors and inducers, shALOX12-transduced cells were infected with DENV and ZIKV and incubated with either the lipid peroxide inhibitor or inducer, ferrostatin and erastin, respectively. In DENV- as well as ZIKV- infected cells, ferrostatin treatment led to a substantial decrease in viral titers in shALOX12 cells (Fig. 5A, B). For ZIKV, ferrostatin treatment only reduced infection in shALOX12 but not in shNT control cells, while for DENV, the decrease was comparable in shALOX12 and in shNT cells. This suggests that ZIKV induces more lipid peroxides than the peroxide scavenger can reduce, and only in combination with the knockdown of ALOX12 is the treatment sufficient to inhibit ZIKV infection. And while erastin led to increased DENV replication in shNT-transduced cells, this was not observed in shALOX12-transduced cells (Fig. 5). Therefore, erastin treatment alone is insufficient to induce threshold levels of lipid peroxides, implying that DENV depends on ALOX12-dependent lipid peroxidation for efficient replication.

### ALOX12-knockdown and ferrostatin treatment block lipid peroxidation in ZIKV-infected cells

**Fig. 6:**
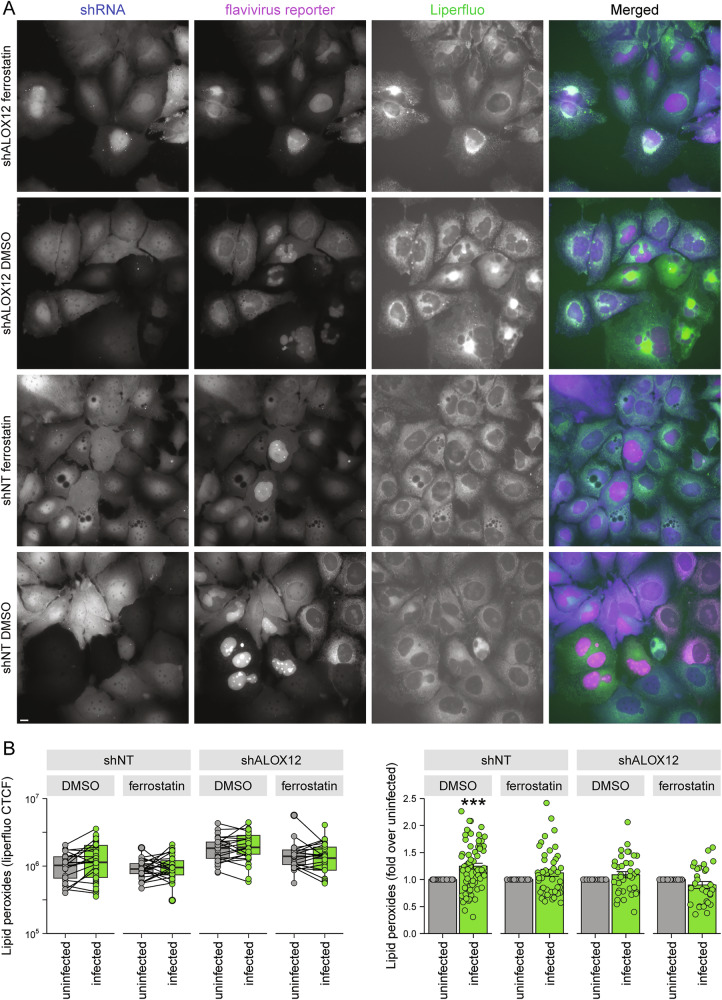
ALOX12 depletion and ferrostatin treatment limit lipid peroxidation in ZIKV-infected cells. **A** Huh7 cells expressing an iRFP-based infection reporter were transduced with lentiviruses carrying shALOX12 or shNT, infected with ZIKV MOI 2, and additionally treated with the ferroptosis inhibitor ferrostatin or DMSO as a control. At 48 hpi, Leibovitz’s medium containing 5 µM Liperfluo was added to the cells, and after 30 min incubation, the cells were analyzed by wide-field fluorescence microscopy. Green fluorescence represents the oxidized form of Liperfluo, while nuclear iRFP localization indicates ZIKV infection and mCherry fluorescence the presence of NT or ALOX12 shRNAs. ZIKV-infected and uninfected control cells in the same image were randomly selected based on the presence of the shRNA marker and the localization of the ZIKV cell-based reporter. Shown are representative images (scale bar 10 µm). **B** Quantification of lipid peroxide levels as corrected total cell fluorescence (CTCF). For the raw data, each line represents paired measurements in one image. Box plots indicate median (center line), upper and lower quartiles (box limits), and interquartile range (whiskers). CTCF values of infected cells were normalized to uninfected cells within the same image (Mean ± SEM, 28–60 cells from 3 independent experiments, ***p ≤ 0.001, two-tailed one-sample Student’s *t*-test).

To determine if ALOX12 contributes to lipid peroxidation in ZIKV infection, we quantified lipid peroxide levels using Liperfluo CTCF in shALOX12- and shNT-transduced cells infected with ZIKV and treated with ferrostatin or DMSO control. In this experimental setup, infected and uninfected cells were compared in the same field of view. Only cells expressing mCherry as a marker for lentiviral transduction and the cell-based infection reporter were used for the analysis (Fig. 6A). In the DMSO control cells, lipid peroxide levels increased significantly in ZIKV-infected shNT cells but not in shALOX12-expressing cells, indicating that ALOX12 depletion reduces lipid peroxide generation (Fig. 6B). Likewise, in ferrostatin-treated cells, ZIKV did not significantly elevate lipid peroxide levels. Of note, only the combination of ALOX12 knockdown and ferrostatin treatment reduced lipid peroxide levels below the levels observed in uninfected neighboring cells, again indicating that this combined intervention is required to lower lipid peroxide levels sufficiently to inhibit ZIKV infection.

### Ferrostatin treatment alters ZIKV replication organelles

**Fig. 7:**
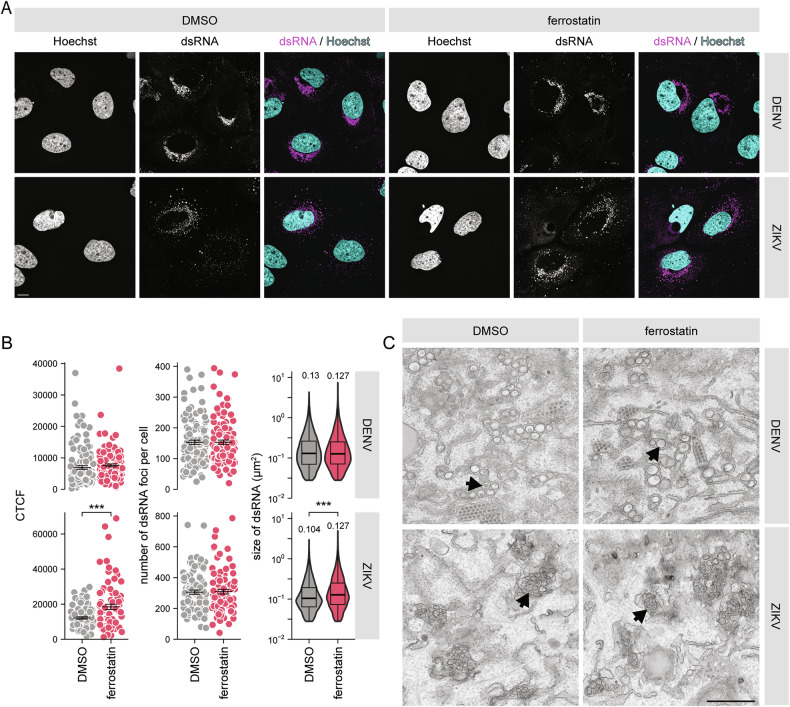
Ferrostatin treatment affects replication organelles. **A**, **B** Huh7 cells were infected and treated with either DMSO or ferrostatin (MOIs DENV: 1, ZIKV: 1). Cells were fixed 48 hpi, stained with antibodies against dsRNA and Hoechst to visualize nuclei, and analyzed by confocal microscopy. Shown are representative images (**A**, scale bar 10 μm) and quantification of dsRNA signal as corrected total cell fluorescence (CTCF) and dsRNA foci number and size using the particle analyzer function of Fiji (27–45 cells per condition from 3 independent experiments, CTCF and number of dsRNA foci: mean ± SEM, ***p ≤ 0.001, unpaired two-tailed Student’s *t*-test, dsRNA foci size: box plots indicate median (center line), upper and lower quartiles (box limits), interquartile range (whiskers), ***p ≤ 0.001, unpaired two-tailed Mann–Whitney *U* test). **C** Infected cells (MOIs DENV: 1, ZIKV: 2) treated with either DMSO or ferrostatin were fixed 48 hpi and processed for EM. Shown are representative images (scale bar = 500 nm). Arrows indicate vesicle packets.

In ALOX12-deficient cells, we observed elevated dsRNA total cell fluorescence (CTCF) and increased dsRNA foci size in immunofluorescence microscopy for DENV and smaller dsRNA foci for ZIKV, while the ultrastructure of vesicle packets was intact (Fig. 2). In order to decipher if ferrostatin treatment may cause similar alterations in dsRNA foci, we treated DENV- and ZIKV-infected Huh7 cells with the ferrostatin and analyzed the cells by immunofluorescence staining with dsRNA antibodies and quantified the dsRNA signal by determining CTCF as well as dsRNA foci number and foci size per cells. DENV infection is impaired in ferrostatin-treated cells, but only a slight, non-significant decrease in dsRNA foci size was detected (Fig. 7A, B). The dsRNA signal appeared to cluster around the nucleus, similar to what we observed in shALOX12-deficient cells, but the much stronger antiviral effect of ferrostatin treatment compared to the ALOX12 knockdown decreased the overall dsRNA signal. ZIKV-infected cells had significantly higher CTCF values and bigger dsRNA foci after ferrostatin treatment as compared to the DMSO control (Fig. 7A, B), which correlates with the increased ZIKV E protein levels after ferrostatin treatment (Fig. 4A). On an ultrastructural level, the replication organelles of both DENV and ZIKV did not appear different between ferrostatin-treated and control cells (Fig. 7C).

### Iron chelation blocks ZIKV and DENV infection

**Fig. 8:**
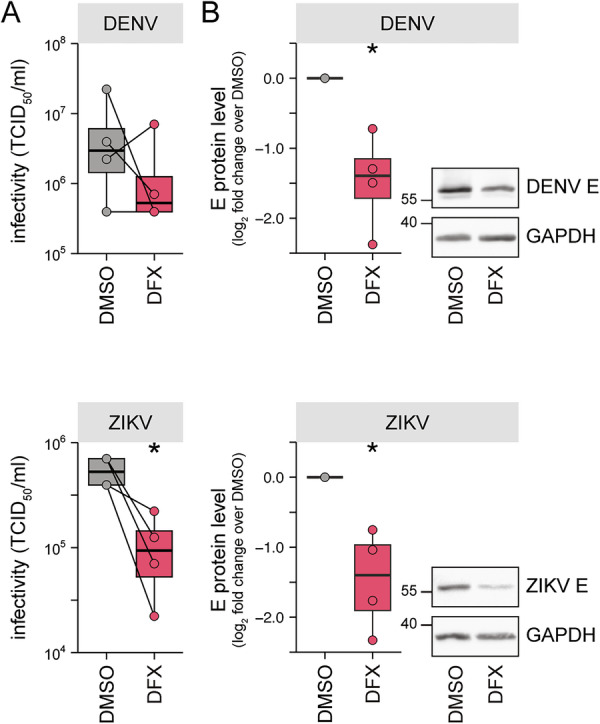
Iron chelation blocks both DENV and ZIKV infection. Huh7 cells were infected with DENV or ZIKV (MOIs DENV: 0.05, ZIKV: 0.1) and treated with the iron chelator deferoxamine-mesylate (DFX) or DMSO as control. **A** Viral titers were determined at 48 hpi by TCID_50_ titration. **B** Viral E protein levels were assessed at 48 hpi by immunoblot analysis followed by densitometric quantification (log_2_ fold change over DMSO-treated controls). GAPDH was used as a loading control. Box plots indicate median (center line), upper and lower quartiles (box limits), interquartile range (whiskers), and outliers (points) (*n* = 4, *p ≤ 0.05, TCID_50_: unpaired two-tailed Mann–Whitney *U* test, immunoblot: two-tailed one-sample Student’s *t*-test).

Lipid peroxides were elevated in ZIKV but not DENV infection, and lipid peroxide scavenging through ferrostatin treatment impaired DENV but not ZIKV infection. Lipid peroxide generation during ferroptosis is dependent on intracellular free iron availability [52, 53]. We next examined the effect of iron chelation on DENV and ZIKV infection. Huh7 cells treated with the iron chelator deferoxamine-mesylate (DFX), which acts upstream of ferrostatin, showed a significant reduction of viral infection, as measured by viral titers and viral E protein levels, for both ZIKV and DENV infections (Fig. 8).

### Ferrostatin treatment of DENV and ZIKV infection in human microglia and differentiated monocytes

**Fig. 9:**
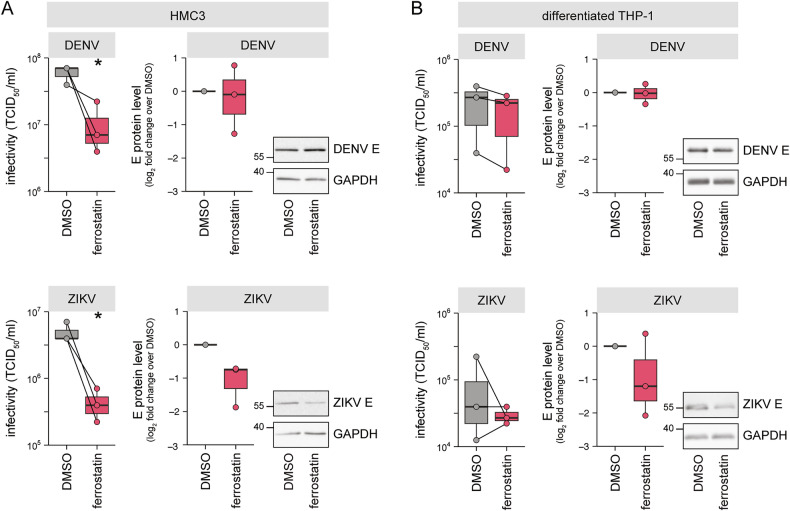
Ferrostatin treatment impairs DENV and ZIKV infection in differentiated THP-1 and immortalized microglia cells. **A** HMC3 cells were infected with DENV or ZIKV (MOIs DENV: 0.1, ZIKV: 0.1) and treated with ferrostatin or DMSO as a control. **B** THP-1 monocytes were differentiated using IL-4 (20 ng/ml) and PMA (20 ng/ml) for 4 days prior to infection. Differentiated THP-1 were infected with DENV or ZIKV (MOIs DENV: 1, ZIKV: 4) and treated with ferrostatin or DMSO as a control. Viral titers were determined at 48 hpi by TCID_50_ titration, and viral E protein levels were assessed at 48 hpi by immunoblot analysis followed by densiometric quantification (log_2_ fold change over DMSO-treated controls). GAPDH was used as a loading control. Box plots indicate median (center line), upper and lower quartiles (box limits), interquartile range (whiskers), and outliers (points) (*n* = 3, *p ≤ 0.05, TCID_50_: unpaired two-tailed Mann–Whitney *U* test, immunoblot: two-tailed one-sample Student’s *t*-test).

Ferrostatin treatment impaired DENV but not ZIKV infection in Huh7 cells (Fig. 4). We next assessed its effect in additional cell types more relevant to orthoflavivirus pathogenesis. Ferrostatin treatment significantly impaired both DENV and ZIKV infection in immortalized human microglia cells (HMC3), as shown by significantly reduced viral titers for both viruses and decreased E protein levels for ZIKV at 48 hpi compared to DMSO-treated controls (Fig. 9A). A slight inhibitory effect was observed in IL-4/PMA-differentiated THP-1 monocytes (Fig. 9B), though the magnitude of reduction was less pronounced and did not reach statistical significance. This likely reflects cell-type-specific differences in ferroptosis sensitivity and viral replication dynamics. Orthoflaviviruses replicate faster and to higher titers in HMC3 compared to in differentiated THP-1 cells.

## Discussion

Lipid mediators are bioactive lipids derived from membrane and storage lipids that regulate key physiological processes, especially inflammation and immune responses. With short half-lives, they are synthesized in response to cellular signals by enzymes such as COX, ALOX, and cytochrome P450, and can promote either pro- or anti-inflammatory effects.

**Fig. 10:**
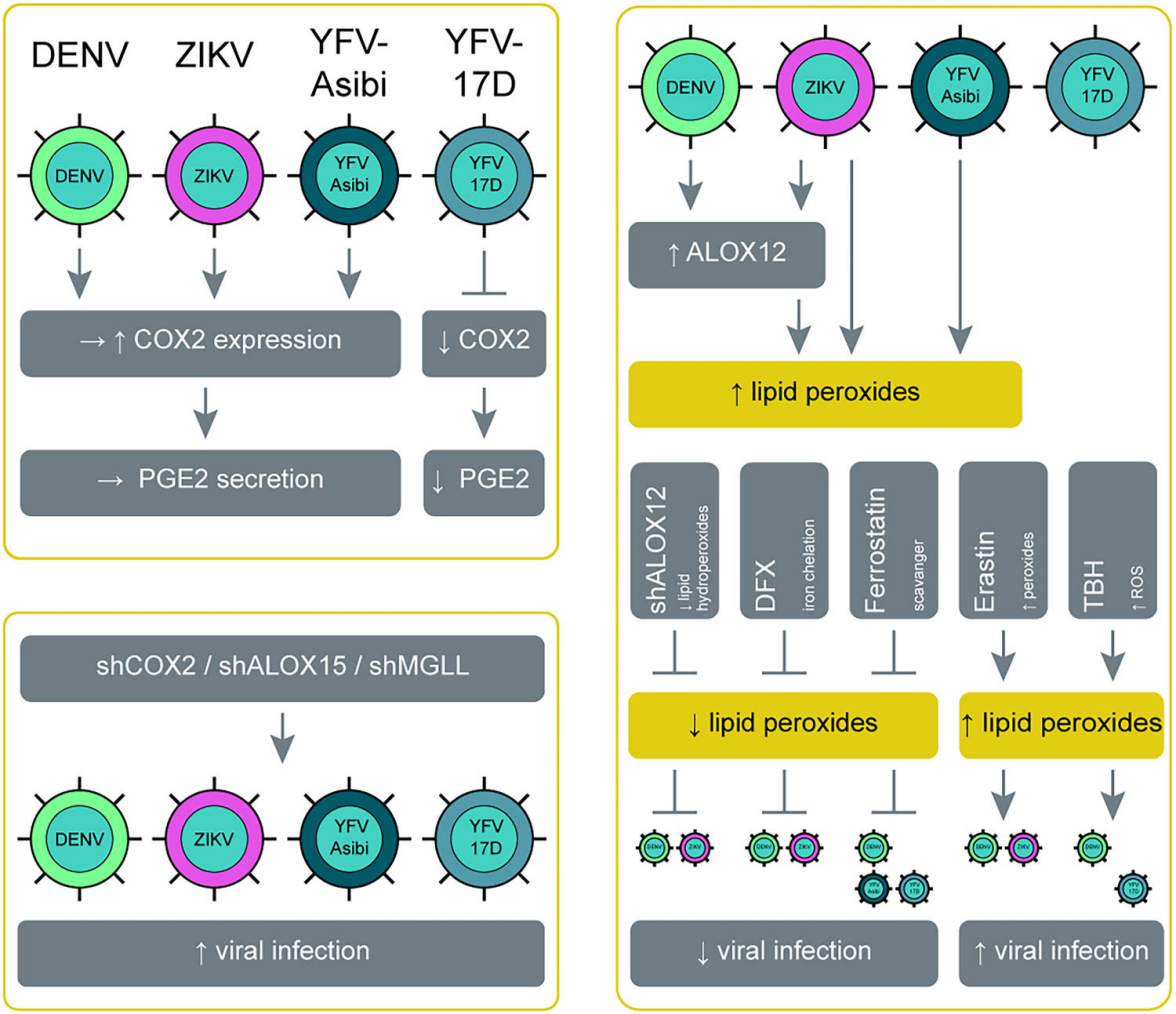
Proposed model of lipid peroxidation during orthoflavivirus infection. See text for details.

Here, four enzymes in the ALOX/COX pathway, namely ALOX5, ALOX12, ALOX15, and COX2, and two lipolytic enzymes, particularly involved in lipid mediator synthesis (MGLL and cPLA2), were examined for their roles in DENV, ZIKV, YFV-Asibi, and YFV-17D infection. Regarding the ALOX/COX pathway, knockdowns of ALOX15 and COX2 had the most significant impact on all orthoflaviviruses examined, as demonstrated by both immunoblot analysis and CPE assays (Fig. 10). The results suggest an anti-flaviviral role for ALOX15 and COX2 in Huh7 cells. This contrasts with findings demonstrating that overexpression of COX2 or exogenous treatment with PGE2 facilitates DENV-2 infection by enhancing the activity of the viral polymerase [30]. But consistent with this study, we also observed that DENV induces the expression of COX2, an effect that was also seen in ZIKV and YFV-Asibi but not YFV-17D infection. This difference between YFV-17D and YFV-Asibi correlated with PGE2 release from infected cells. YFV-17D, a live-attenuated vaccine strain, differs from the wild-type Asibi strain by just 20 amino acids, including 12 mutations in the E protein that mediate clathrin-independent entry, contrasting the classical clathrin-mediated pathway used by Asibi [54]. These E protein mutations enhance binding, uptake, and RNA delivery, making YFV-17D more efficient at infecting human cells. This is consistent with our finding that similar infectivity levels at 48–72 hpi were achieved using a much lower MOI for YFV-17D compared to YFV-Asibi. The clathrin-independent entry of YFV-17D may lead to differential exposure and a distinct innate immune response profile, potentially including suppression of COX2 via pathways activated downstream of alternative pattern recognition receptors (e.g., in late endosomes). This could account for the uniquely suppressed COX2/PGE2 axis observed with this strain. It was previously shown that hepatocytes infected with YFV-17D exhibited a more pronounced immune response, producing significantly higher concentrations of both pro- and anti-inflammatory cytokines compared to those infected with the YFV-Asibi [55]. Notably, IL-4 and IL-6 release were significantly higher in YFV-17D-infected cells than in YFV-Asibi-infected cells, which could serve to limit infection progression and potentially reduce disease severity [56]. In addition, YFV-17D infection led to higher expression of antiviral cytokines such as IFN-γ, IL-29, ISG56, CCL5, and CXCL10 compared to YFV-Asibi infection. This suggests that the entry mechanism of YFV-17D is associated with a more robust cytokine-mediated antiviral response [55]. It is likely that the differential immunogenicity between the two strains contributes to the difference in pathogenicity.

In addition, DENV and ZIKV were shown to increase ALOX12 expression, an enzyme that, in addition to its function in lipid mediator synthesis, has been linked to lipid peroxidation and ferroptosis [27]. Orthoflavivirus infection itself caused an accumulation of oxidized lipids, with ZIKV infection having the strongest effect. Inducing lipid peroxidation and ferroptosis *via* treatment with erastin, which blocks GPX4-mediated detoxification of lipid hydroperoxides to non-toxic lipid alcohols, fuels both DENV and ZIKV infection but has minor antiviral effects on both YFV strains. These results indicate a profound difference between DENV and ZIKV on the one hand, and YFV on the other hand, in their interplay with lipid peroxides and ALOX12.

In our study, ALOX12 emerged as a major contributor to lipid peroxide generation during DENV and ZIKV infection. In ZIKV-infected cells, only the combination of ALOX12 knockdown and treatment with the lipid peroxide scavenger ferrostatin reduced lipid peroxide levels below the levels of uninfected neighboring cells. Furthermore, ALOX12 knockdown resulted in significantly reduced dsRNA foci size, whereas treatment with ferrostatin, which did not impair ZIKV infection, increased dsRNA foci size as well as viral E protein level. These seemingly contradictory findings may suggest a compensatory mechanism in which lipid peroxide scavenger treatment promotes ALOX12-mediated lipid peroxide production to counteract the scavenger-treatment effects. Consequently, sustained lipid peroxide generation may facilitate viral replication, leading to enhanced dsRNA accumulation, enlarged dsRNA foci, and increased viral protein levels. Consequently, upstream iron chelation blocked ZIKV replication, further highlighting the beneficial effect of lipid peroxidation on ZIKV infection.

In contrast, DENV infection did not cause measurable accumulation of lipid peroxides, and its replication was boosted by treatment with ROS inducers. ALOX12-knockdown mitigated the proviral effect of erastin, implicating a complete dependence of DENV on ALOX12-dependent lipid peroxidation to initiate lipid peroxidation-driven processes. Like ZIKV, DENV is sensitive to iron chelation. However, treatment of ALOX12-knockdown cells with ferrostatin still decreased, and TBH-mediated ROS increased DENV infection, indicating that DENV benefits from additional oxidative stress for efficient replication. Moreover, in DENV-infected ALOX12-deficient cells, dsRNA staining exhibited a more clustered distribution and increased CTCF levels compared to control cells. However, despite this increased dsRNA accumulation, ALOX12 knockdown reduced DENV titers and E protein levels. These findings suggest that ALOX12 depletion causes aberrant ROs. The clustering of dsRNA foci may reflect the loss of ALOX12-driven lipid peroxidation, which impairs membrane dynamics at replication organelles, causing them to accumulate as stalled, non-productive structures.

Regarding our investigations into other cell types, both DENV and ZIKV were impacted by ferrostatin treatment in human microglia cells and, to a smaller extent, differentiated THP-1 cells, indicating differences in ferroptosis sensitivity compared to the hepatoma cells. Indeed, neither cell type tolerated erastin treatment (data not shown), preventing us from analyzing viral replication under ferroptosis induction. While cell-type-specific sensitivities to lipid peroxidation can be observed, the proviral activity is apparent across the cell lines examined. This is also in line with other previous studies, which showed DENV and ZIKV impacting oxidative stress in infected cells. DENV induces ER stress and autophagy, leading to elevated ROS levels at late stages of infection, a process that is critical for efficient replication [57]. In stem cell–derived human astrocytes, ZIKV infection increases ROS levels through mitochondrial dysfunction, a phenotype that may be related to the neurological disorders observed in ZIKV-infected patients [58]. Similarly, ZIKV infection induces ROS, lipid peroxidation, and protein carbonylation products in human glioblastoma and hepatoma cells and brain and liver tissue of infected mice [59].

Our results highlight the virus-specific interaction of orthoflaviviruses with a cellular cell death pathway that fuels virus replication and point towards differences in lipid COX2-dependent PGE2 production between wild-type viruses and the YFV vaccine strain. But why reactive oxygen species and lipid peroxidation are proviral for orthoflaviviral replication, and the molecular mechanisms that lead to elevated lipid peroxide levels have yet to be determined.

## Supplementary information

Supplementary Information

Original data

## Data Availability

All data needed to evaluate the conclusions in the paper are present in the paper and/or the Supplementary Materials.
