# Supplementary material for: Lipid peroxidation impacts orthoflavivirus infection in a virus-dependent manner
Source: Cell Death Dis. 2026 Jul 8;17(1):627. doi: 10.1038/s41419-026-09071-8 (PMC13346785; doi:10.1038/s41419-026-09071-8)
Supplement: Supplementary file 1 — Supplementary Information [file 41419_2026_9071_MOESM1_ESM.pdf]

Supplementary Information

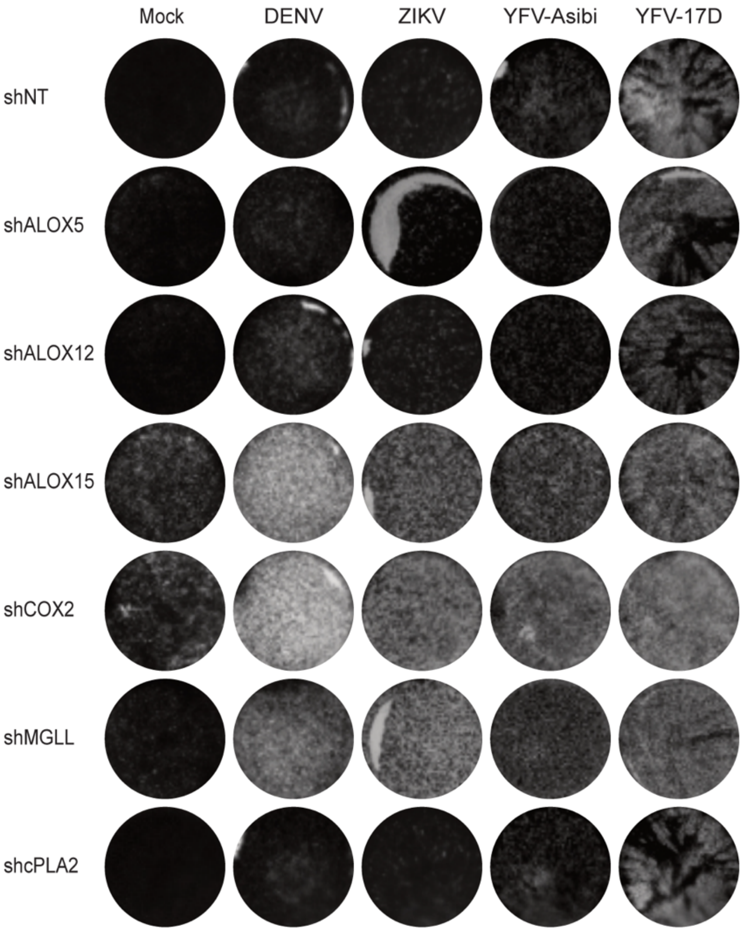

Figure S1: Knockdown of ALOX15, COX2, and MGLL increases CPE after orthoflavivirus infection.
shRNA-transduced Huh7 cells were infected with different orthoflavivirus stocks (MOIs DENV: 0.02, ZIKV: 0.03, YFV-Asibi: 0.015, YFV-17D: 0.002). Cells were fixed at 3 dpi and stained with crystal violet to visualize the cytopathic effect (CPE) in the cell monolayer.

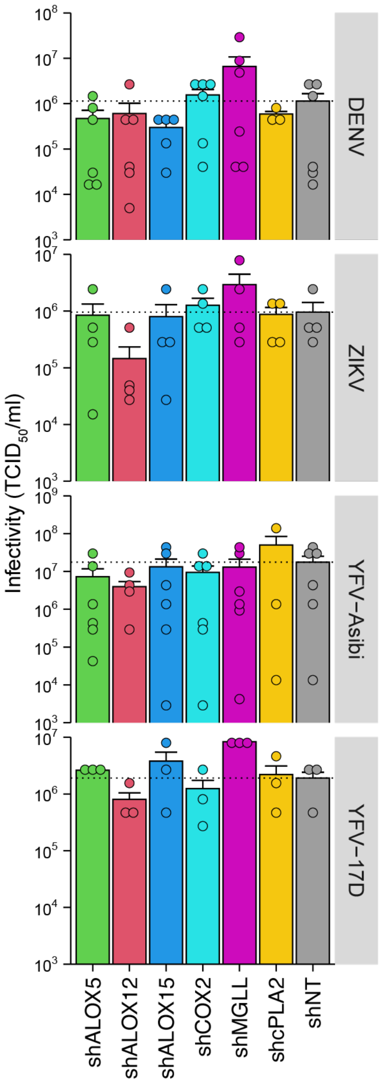

Figure S2: Effects of lipid mediator enzyme knockdown on orthoflavivirus particle production.
shRNA-transduced Huh7 cells were infected at 4 dpt with the different orthoflavivirus (DENV MOI = 0.05, ZIKV MOI = 0.1, YFV-Asibi = 0.015, YFV-17D MOI= 0.002), virus titers were determined by $TCID_{50}$ assays (Mean + SEM, n = 3–6).

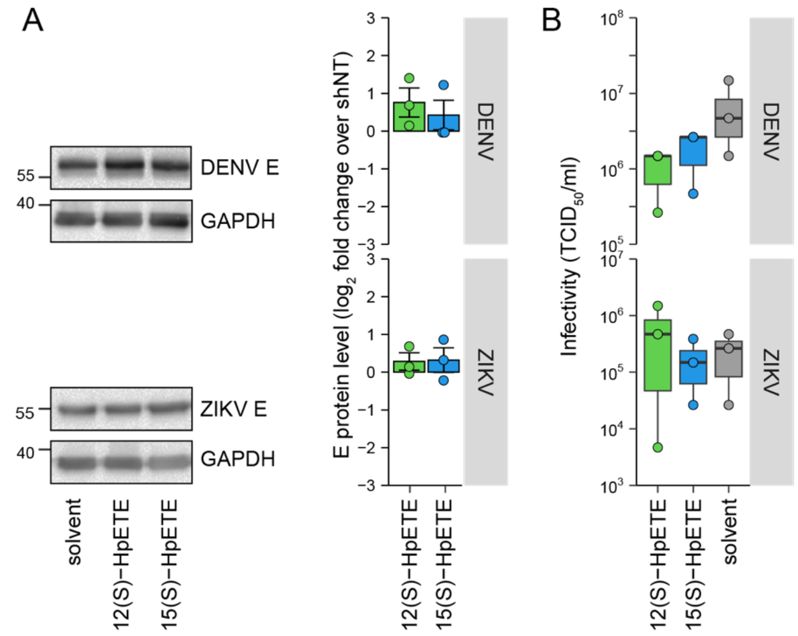

Figure S3: Effects of lipid mediators 12(S)-HpETE and 15(S)-HpETE on DENV and ZIKV infection.

Huh7 cells were infected with DENV and ZIKV (DENV: 0.02, ZIKV: 0.03) and supplemented with12(S)-HpETE and 15(S)-HpETE. (A) Viral E protein levels were assessed at 48 hpi by immunoblot analysis followed by densiometric quantification. GAPDH was used as loading control. Shown is the signal intensity normalized to GAPDH as log_2 fold change over shNT (Mean ± SEM, n = 3, two-tailed one sample Student's *t*-test). (B) Viral titers were determined at 48 hpi by $TCID_{50}$ titration. Box plots indicate median (center line), upper and lower quartiles (box limits), interquartile range (whiskers), and outliers (points) (n = 3, unpaired two-tailed Mann–Whitney U test).
