# Supplementary material for: Lipid peroxidation impacts orthoflavivirus infection in a virus-dependent manner
Source: Cell Death Dis. 2026 Jul 8;17(1):627. doi: 10.1038/s41419-026-09071-8 (PMC13346785; doi:10.1038/s41419-026-09071-8)
Supplement: Supplementary file 2 — Original data [file 41419_2026_9071_MOESM2_ESM.pdf]

**Uncropped immunoblots**

PageRuler Prestained Protein Ladder (#26616 Thermo Fisher Scientific)

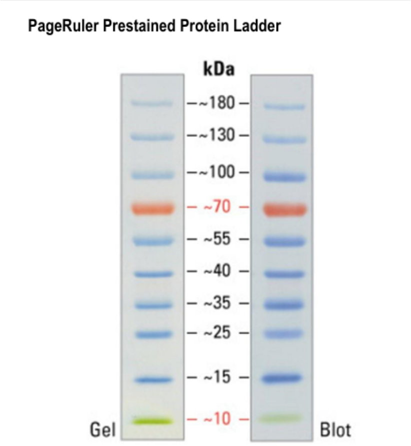


| **Figure 1F** | |
|---|---|
| FlaviE – DENV infected KD cells<br><br>shALOX5- shALOX12-shALOX15-shCOX2-shMGLL-shcPLA2-shNT<br><br>20240409 | 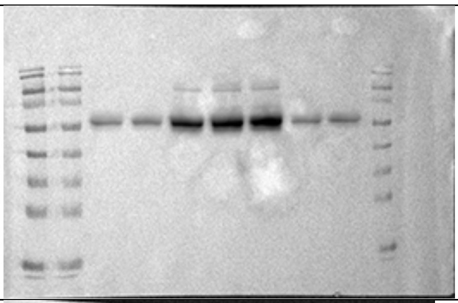 |
| GAPDH – DENV infected KD cells<br><br>shALOX5- shALOX12-shALOX15-shCOX2-shMGLL-shcPLA2-shNT | 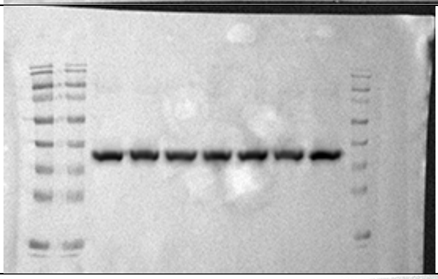 |
| FlaviE- ZIKV infected KD cells<br><br>shALOX5- shALOX12-shALOX15-shCOX2-shMGLL-shcPLA2-shNT<br><br>20240409 | 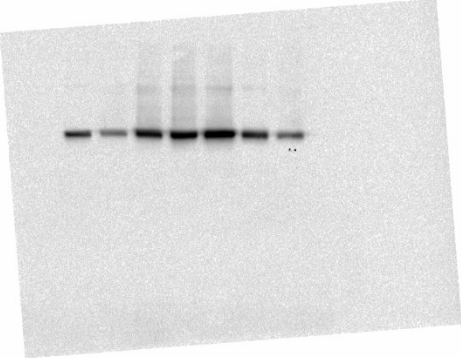 |

| | |
|---|---|
| GAPDH- ZIKV infected KD cells<br><br>shALOX5- shALOX12-shALOX15-shCOX2-shMGLL-shcPLA2-shNT | 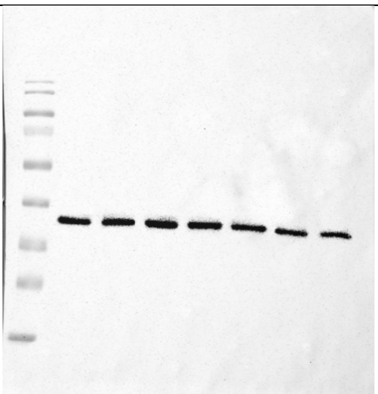 |
| FlaviE- YFV-Asibi infected KD cells<br><br>shALOX5- shALOX12-shALOX15-shCOX2-shMGLL-shcPLA2-shNT<br><br>20240609 | 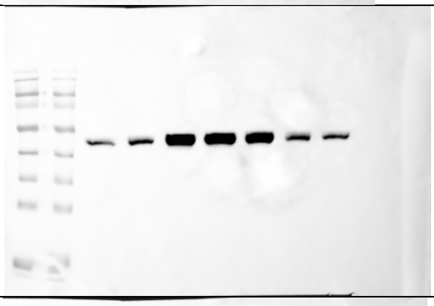 |
| GAPDH- YFV-Asibi infected KD cells<br><br>shALOX5- shALOX12-shALOX15-shCOX2-shMGLL-shcPLA2-shNT<br><br>20240609 | 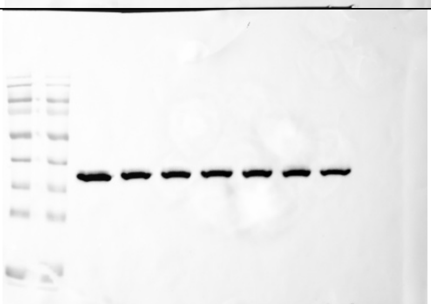 |
| FlaviE- YFV-17D infected KD cells<br><br>shALOX5- shALOX12-shALOX15-shCOX2-shMGLL-shcPLA2-shNT<br><br>20241018 | 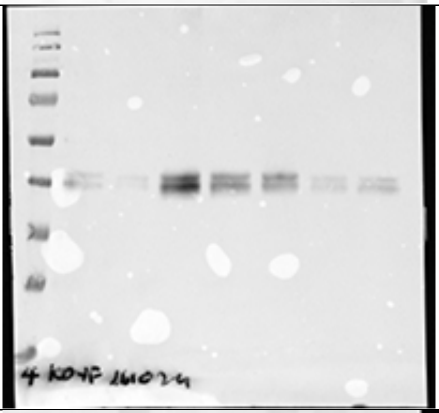 |
| GAPDH- YFV-17D infected KD cells<br><br>shALOX5- shALOX12-shALOX15-shCOX2-shMGLL-shcPLA2-shNT<br><br>20241018 | 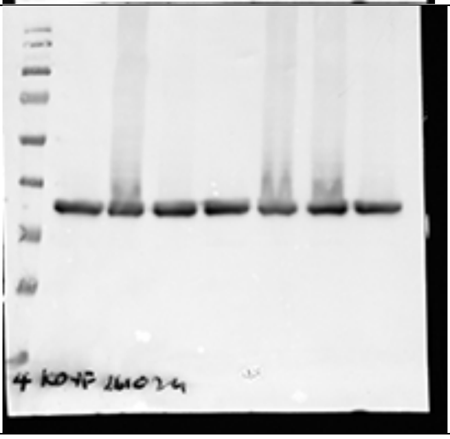 |
| **Figure 3C** | |

| | |
|---|---|
| FlaviE – orthoflavivirus-infected treated with TBH<br>DENV- YFV-Asibi- **ZIKV**- YFV-17D Mock TBH+<br><br>20241017 | 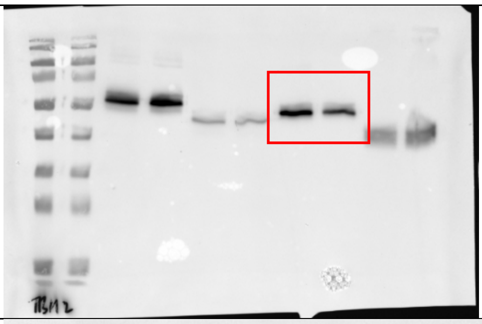 |
| GAPDH – orthoflavivirus-infected treated with TBH<br>DENV- YFV-Asibi- **ZIKV**- YFV-17D Mock TBH+ | 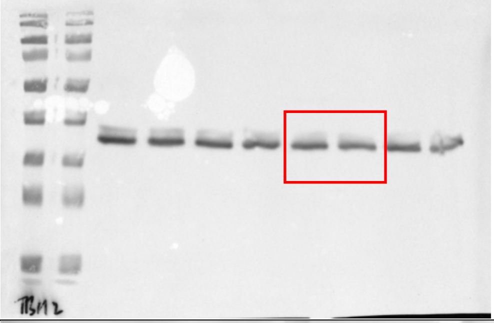 |
| FlaviE – orthoflavivirus-infected treated with TBH<br>DENV- YFV-Asibi- ZIKV- **YFV-17D** Mock TBH+<br><br>20241017 | 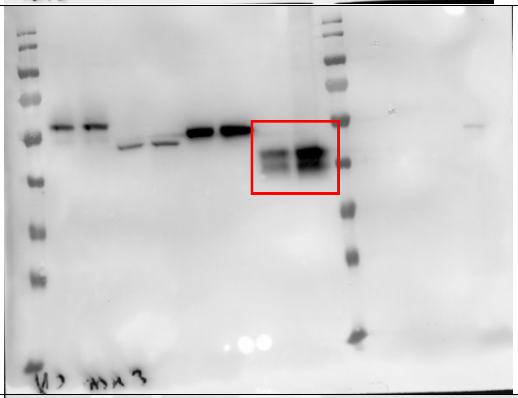 |
| GAPDH – orthoflavivirus-infected treated with TBH<br>DENV- YFV-Asibi- ZIKV- **YFV-17D** Mock TBH+ | 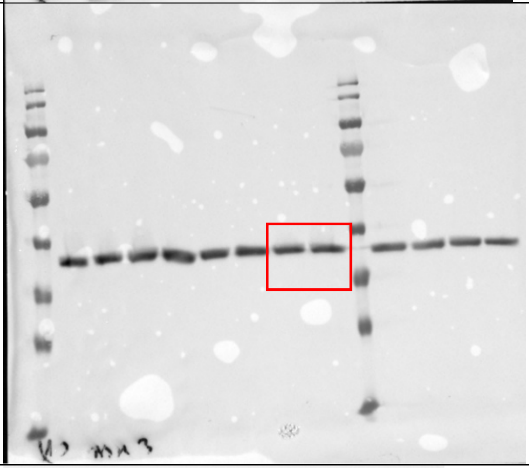 |
| FlaviE - **YFV-Asibi** infected treated with TBH<br>Mock TBH+<br>Difference MOI | 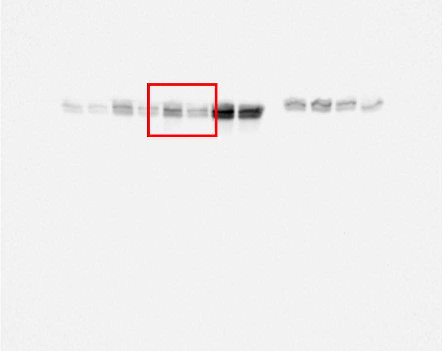 |

| | |
|---|---|
| GAPDH - **YFV-Asibi** infected treated with TBH<br><br>Different MOIs | 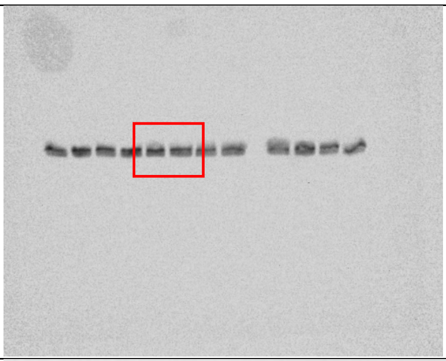 |
| FlaviE – **DENV** infected treated with TBH<br><br>Mock TBH+<br><br>20241010 | 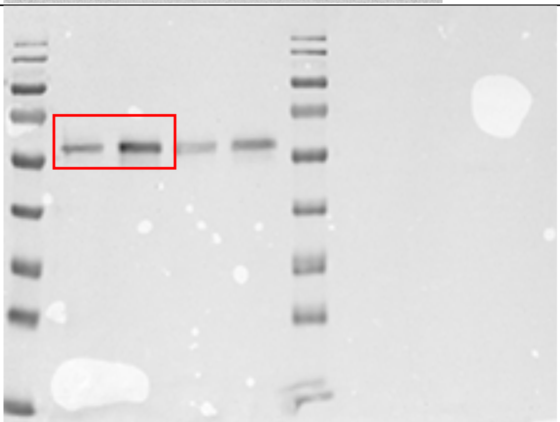 |
| GAPDH – **DENV** infected treated with TBH<br><br>Mock TBH+ | 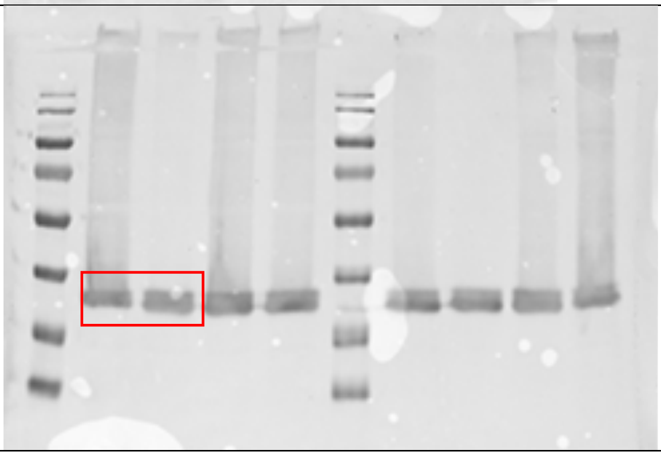 |
| **Figure 4** | |
| FlaviE – DENV infected Huh7, treated with ferrostatin<br><br>DMSO- ferrostatin<br><br>20240317<br><br>**Figure 4B** | 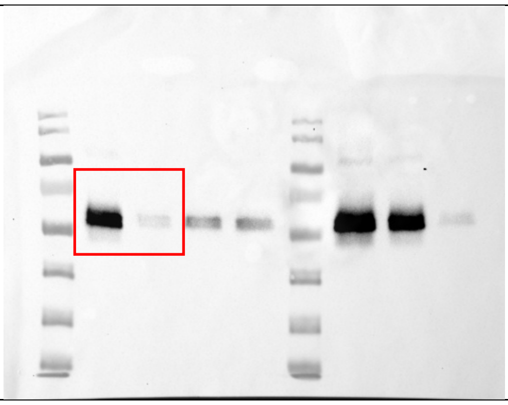 |

| | |
|---|---|
| GAPDH – DENV infected Huh7, treated with ferrostatin<br><br>DMSO- ferrostatin | 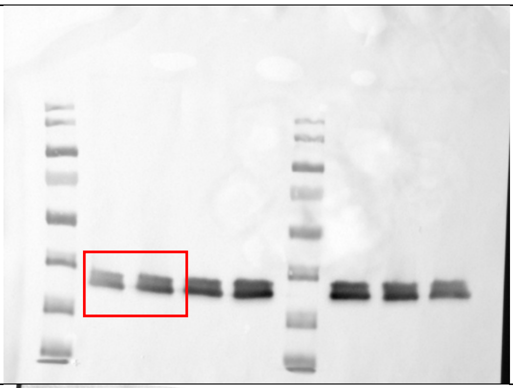 |
| FlaviE – ZIKV infected Huh7, treated with ferrostatin, erastin<br><br>DMSO- ferrostatin – DMSO – erastin<br><br>20241030<br><br>**Figure 4B, D** | 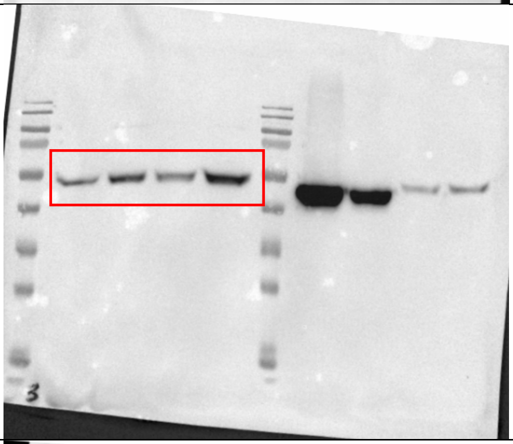 |
| GAPDH – ZIKV infected Huh7, treated with ferrostatin, erastin<br><br>DMSO- ferrostatin – DMSO – erastin<br><br>20241030<br><br>**Figure 4B, D** | 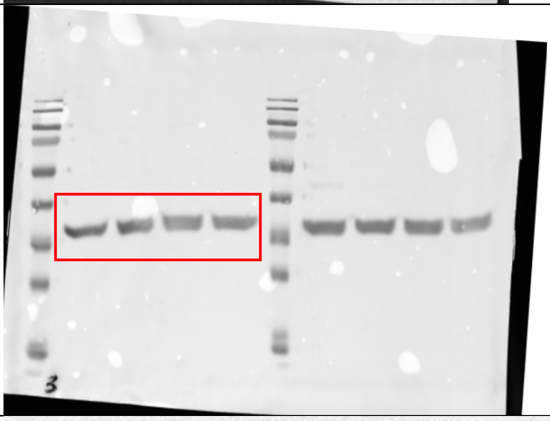 |
| FlaviE – YFV-Asibi infected Huh7, treated with ferrostatin<br><br>DMSO- ferrostatin<br><br>20240506<br><br>**Figure 4B** | 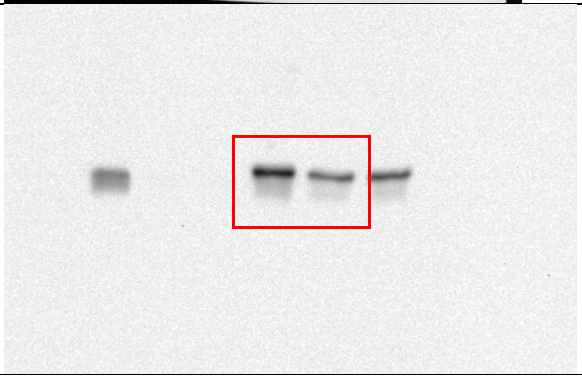 |

| | |
|---|---|
| GAPDH – YFV-Asibi infected Huh7, treated with ferrostatin<br><br>DMSO- ferrostatin<br><br>20240506 | 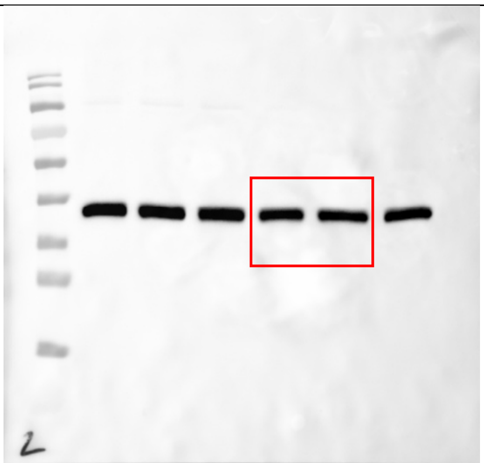 |
| FlaviE – YFV-17D infected Huh7, treated with ferrostatin, erastin<br><br>DMSO- ferrostatin – DMSO – erastin<br><br>20241030<br><br>**Figure 4B, D** | 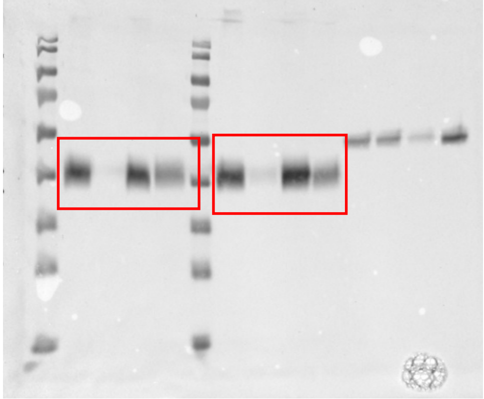 |
| FlaviE – YFV-17D infected Huh7, treated with ferrostatin, erastin<br><br>DMSO- ferrostatin – DMSO – erastin<br><br>20241030<br><br>**Figure 4B, D** | 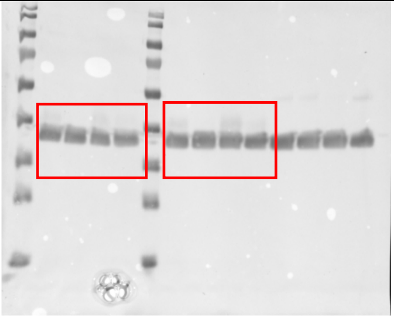 |
| FlaviE – DENV infected Huh7, treated with erastin<br><br>DMSO- erastin<br><br>20240630<br><br>**Figure 4D** | 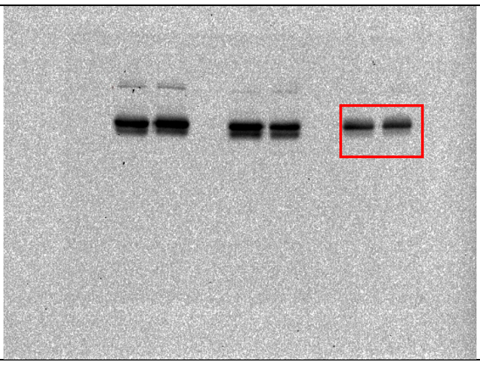 |

| | |
|---|---|
| GAPDH – DENV infected Huh7, treated with erastin<br><br>DMSO- erastin<br><br>20240630 | 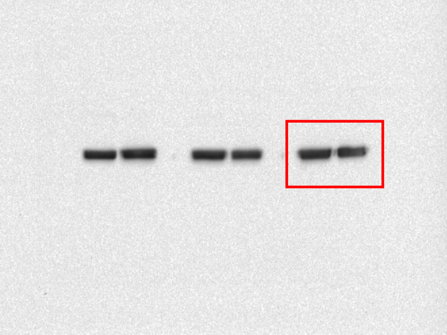 |
| FlaviE – DENV infected Huh7, treated with erastin<br><br>DMSO- erastin<br><br>20240627<br><br>**Figure 4D** | 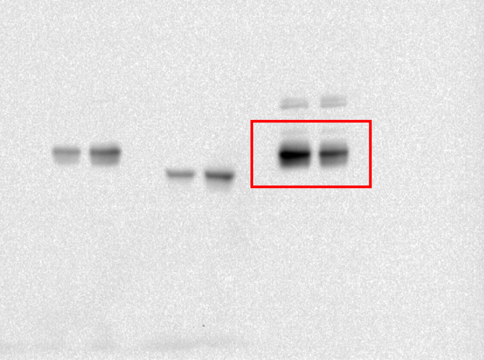 |
| GAPDH – DENV infected Huh7, treated with erastin<br><br>DMSO- erastin<br><br>20240627 | 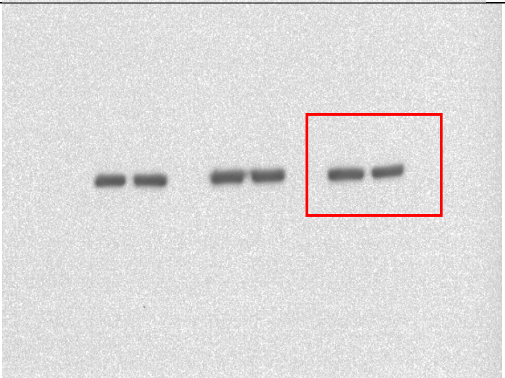 |
| **Figure 5** | |
| FlaviE – DENV infected shNT, ALOX12, treated with ferrostatin<br><br>DMSO- ferrostatin<br><br>shNT- shALOX12<br>20240622<br><br>**Figure 5B** | 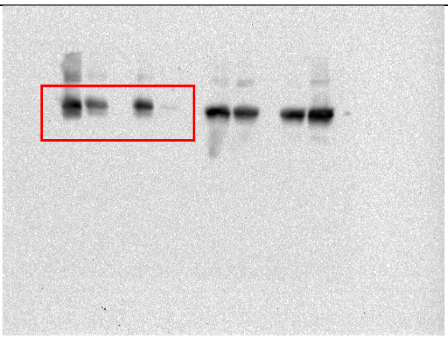 |
| GAPDH – DENV infected shNT, ALOX12 , treated with ferrostatin<br><br>DMSO- ferrostatin<br>shNT- shALOX12<br><br>20240622 | 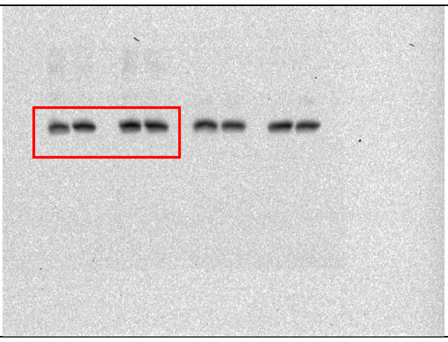 |

| | |
|---|---|
| FlaviE – ZIKV infected shNT treated with ferrostatin, erastin<br><br>DMSO- ferrostatin DMSO - erastin<br><br>shNT<br>202410110<br>**Figure 5 B,D** | 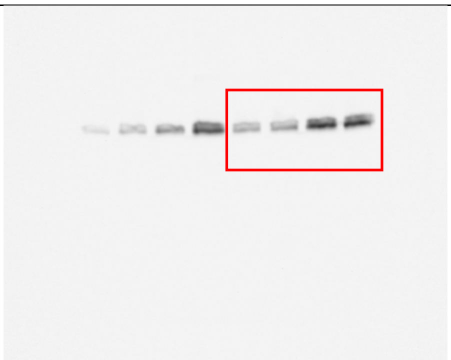 |
| GAPDH – ZIKV infected shNT treated with ferrostatin, erastin<br><br>DMSO- ferrostatin DMSO - erastin<br><br>shNT<br>202410110<br><br>**Figure 5 B,D** | 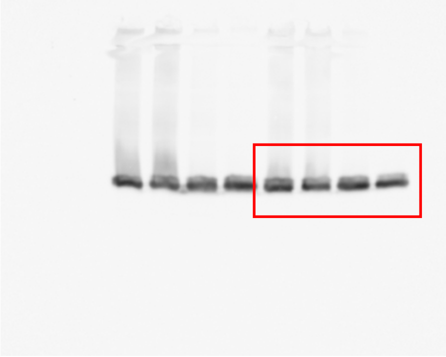 |
| FlaviE – ZIKV infected shALOX12, treated with ferrostatin<br><br>DMSO – ferrostatin<br><br>20241010<br><br>**Figure 5B** | 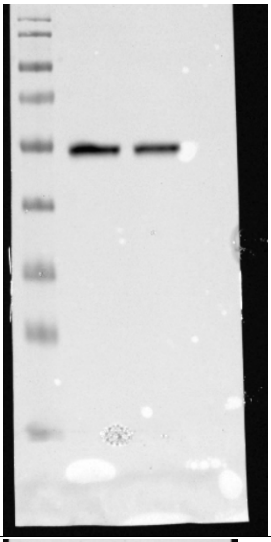 |
| GAPDH – ZIKV infected shALOX12, treated with ferrostatin<br><br>DMSO – ferrostatin<br><br>20241010 | 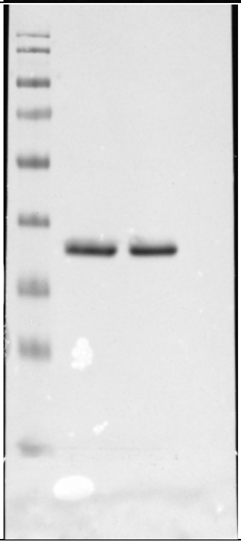 |

| | |
|---|---|
| FlaviE- DENV infected shNT, treated with erastin<br><br>DMSO erastin<br><br>20240629<br><br>**Figure 5D** | 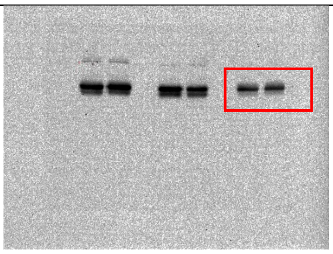 |
| GAPDH - DENV infected shNT, treated with erastin<br><br>DMSO erastin<br><br>20240629 | 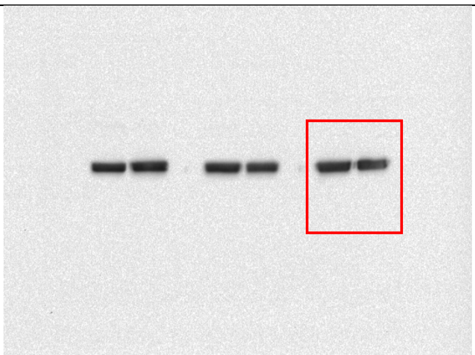 |
| FlaviE- DENV infected shALOX12, treated with erastin<br><br>DMSO erastin<br><br>20240629<br><br>**Figure 5D** | 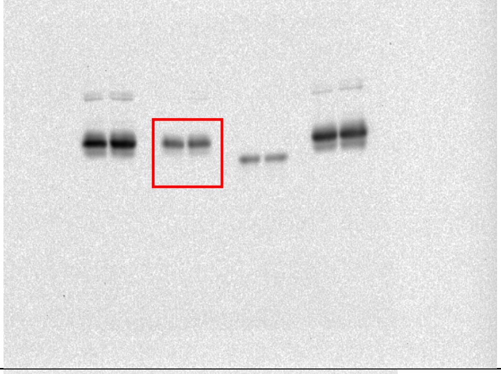 |
| GAPDH- DENV infected shALOX12, treated with erastin<br><br>DMSO erastin<br><br>20240629 | 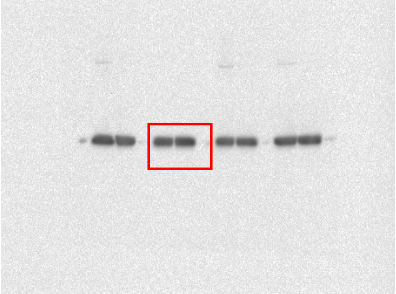 |
| FlaviE- ZIKV infected shALOX12, treated with erastin<br><br>DMSO erastin<br><br>20241010<br><br>**Figure 5D** | 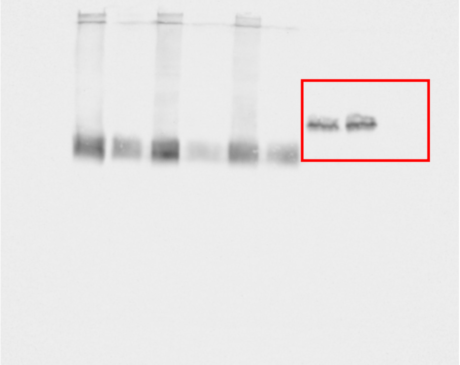 |

| | |
|---|---|
| GAPDH- ZIKV infected shALOX12, treated with erastin<br><br>DMSO erastin<br><br>20241010 | 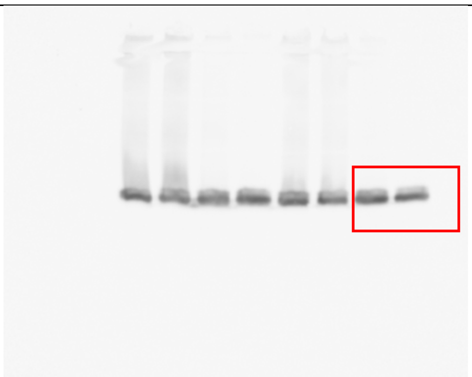 |
| **Figure S3** | |
| FlaviE – DENV infected Huh7 cells supplemented with lipid mediators (Ethanol- 12S/HETE-15S/HETE)<br><br>20240216 | 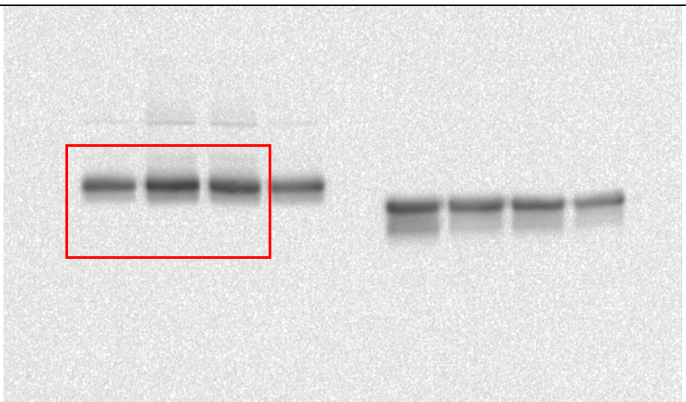 |
| GAPDH – DENV infected Huh7 cells supplemented with lipid mediators (Ethanol- 12S/HETE-15S/HETE)<br><br>20240216 | 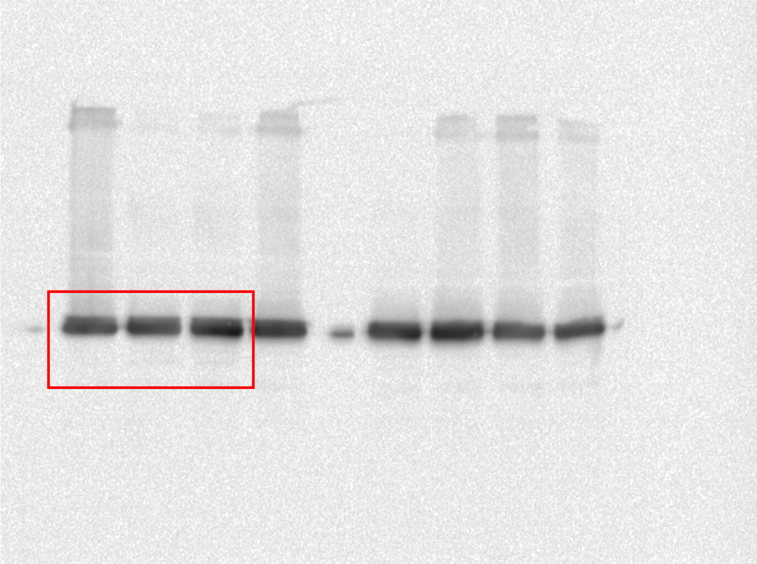 |
| FlaviE – YFV-Asibi infected Huh7 cells supplemented with lipid mediators (Ethanol- 12S/HETE-15S/HETE)<br><br>20240216 | 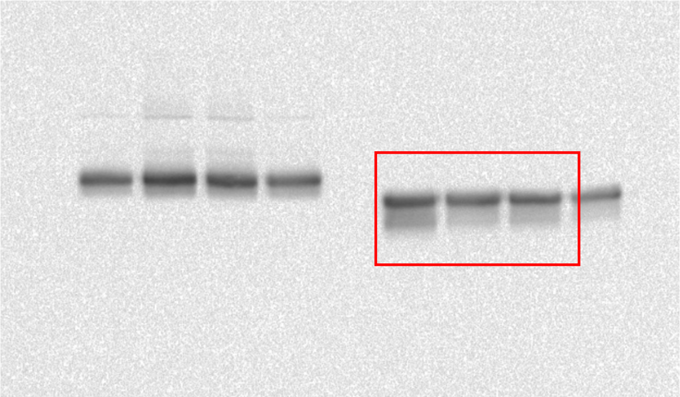 |

| | |
|---|---|
| GAPDH – YFV-Asibi infected Huh7 cells supplemented with lipid mediators (Ethanol- 12S/HETE- 15S/HETE)<br><br>20240216 | 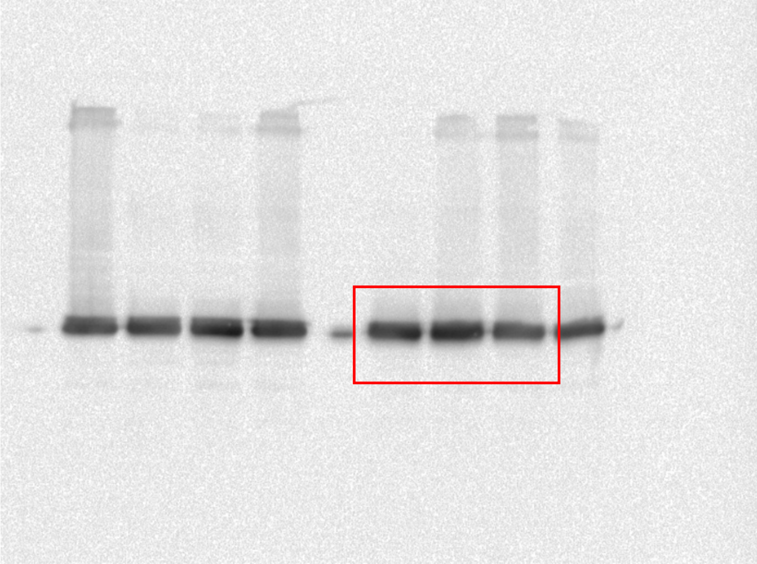 |
| FlaviE – ZIKV infected Huh7 cells supplemented with lipid mediators (Ethanol- 12S/HETE- 15S/HETE)<br><br>20240216 | 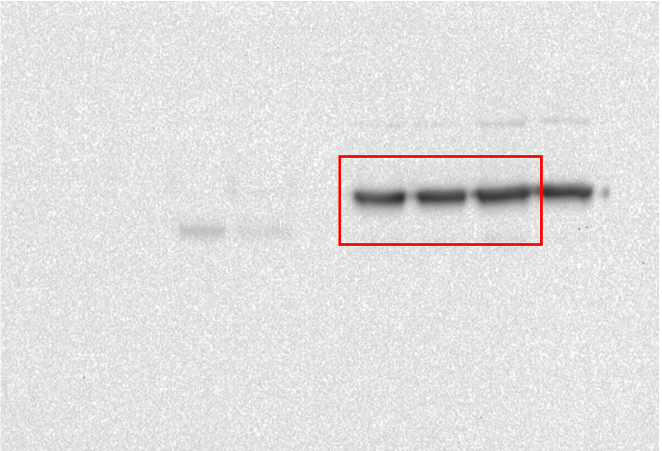 |
| GAPDH – ZIKV infected Huh7 cells supplemented with lipid mediators (Ethanol- 12S/HETE- 15S/HETE)<br><br>20240216 | 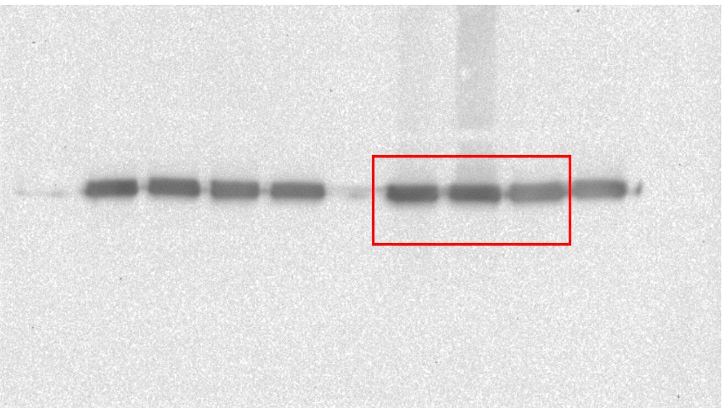 |

| | |
|---|---|
| FlaviE – YFV-17D infected Huh7 cells supplemented with lipid mediators (15S/HETE - 12S/HETE- Ethanol)<br><br>20240324 | 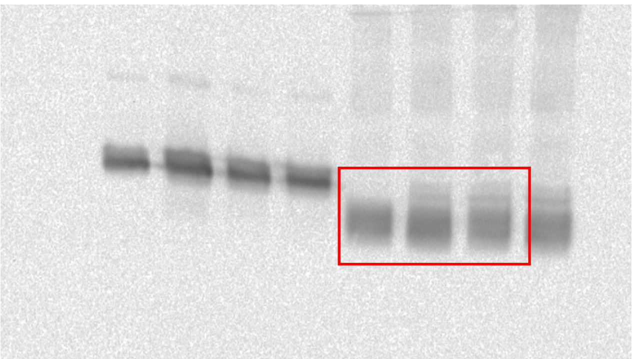 |
| GAPDH – YFV-17D infected Huh7 cells supplemented with lipid mediators (15S/HETE - 12S/HETE- Ethanol)<br><br>20240324 | 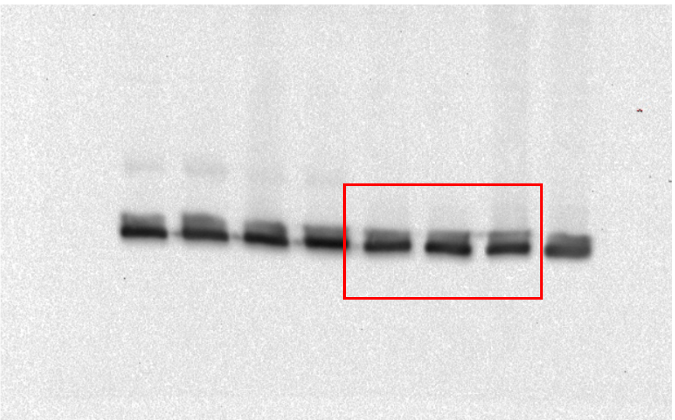 |
| **Figure 8B** | |
| FlaviE – ZIKV infected Huh7 cells treated with DFX ZIKV+DFX – ZIKV-DFX<br><br>20260506 | 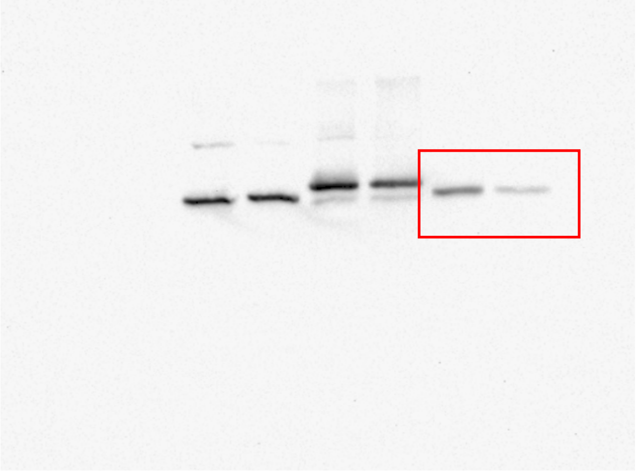 |
| GAPDH – ZIKV infected Huh7 cells treated with DFX ZIKV+DFX – ZIKV-DFX<br><br>20260506 | 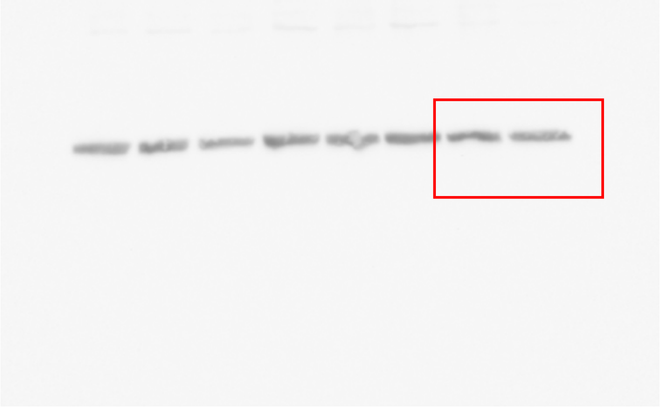 |

| | |
|---|---|
| FlaviE – DENV infected Huh7 cells treated with DFX<br>DENV+DFX – DENV-DFX<br><br>20260506 | 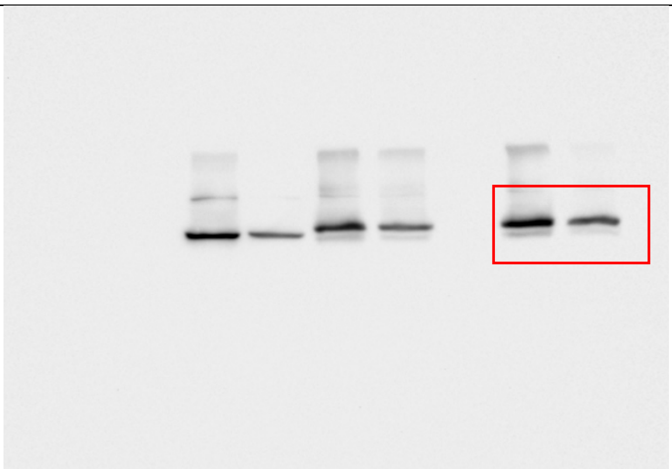 |
| GAPDH – DENV infected Huh7 cells treated with DFX<br>DENV+DFX – DENV-DFX<br><br>20260506 | 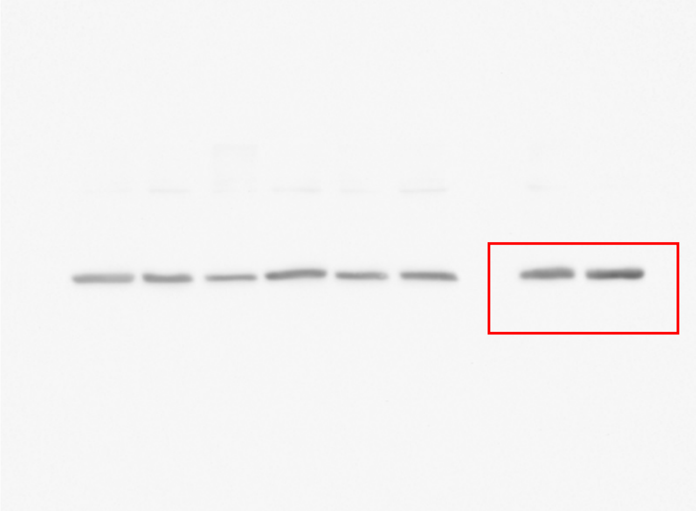 |
| **Figure 9** | |
| FlaviE – ZIKV infected differentiated THP1 cells treated with ferrostatin<br>ZIKV+DMSO – ZIKV+ferrostatin<br><br>20260520<br><br>**Figure 9A** | 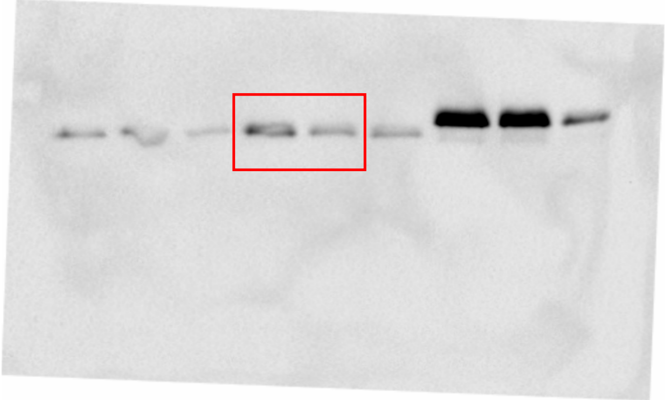 |

| | |
|---|---|
| GAPDH – ZIKV infected differentiated THP1 cells treated with ferrostatin ZIKV+DMSO – ZIKV+ferrostatin<br><br>20260520 | 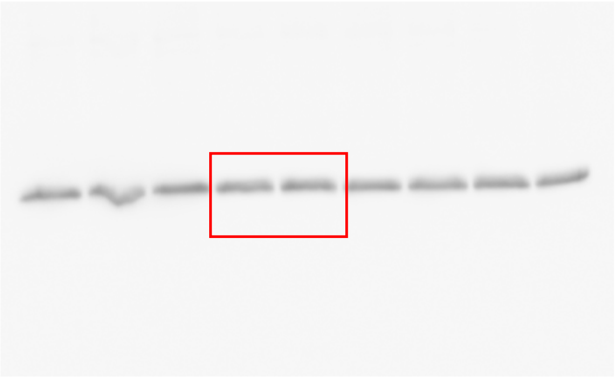 |
| FlaviE –DENV infected differentiated THP1 cells treated with ferrostatin DENV+DMSO – DENV+ferrostatin<br><br>20260520<br><br>**Figure 9A** | 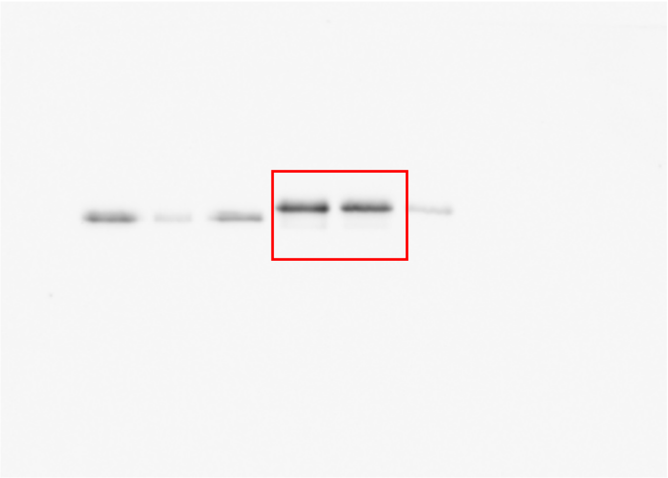 |
| GAPDH –DENV infected differentiated THP1 cells treated with ferrostatin DENV+DMSO – DENV+ferrostatin<br><br>20260520 | 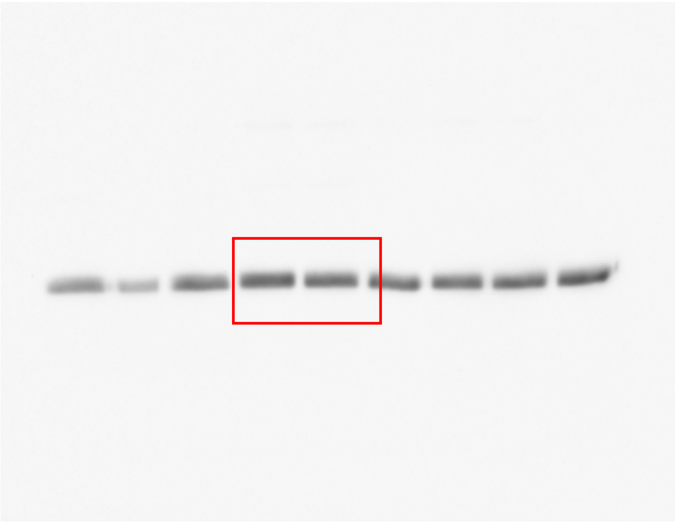 |

| | |
|---|---|
| FlaviE – ZIKV infected HMC3 cells treated with ferrostatin ZIKV+DMSO – ZIKV+ferrostatin<br><br>20260414<br><br>**Figure 9B** | 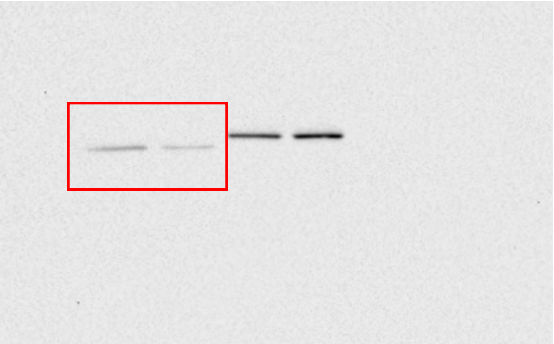 |
| GAPDH – ZIKV infected HMC3 cells treated with ferrostatin ZIKV+DMSO – ZIKV+ferrostatin<br><br>20260414 | 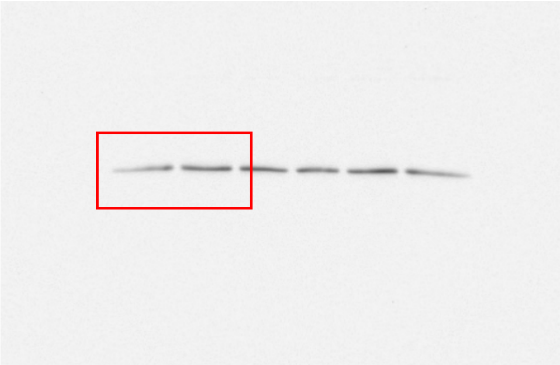 |
| FlaviE – DENV infected HMC3 cells treated with ferrostatin ZIKV+DMSO – ZIKV+ferrostatin<br><br>20260414<br><br>**Figure 9B** | 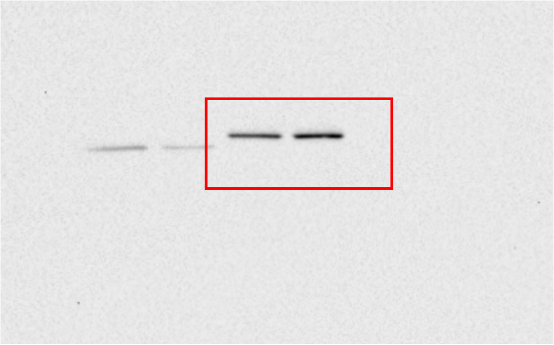 |
| GAPDH – DENV infected HMC3 cells treated with ferrostatin ZIKV+DMSO – ZIKV+ferrostatin<br><br>20260414 | 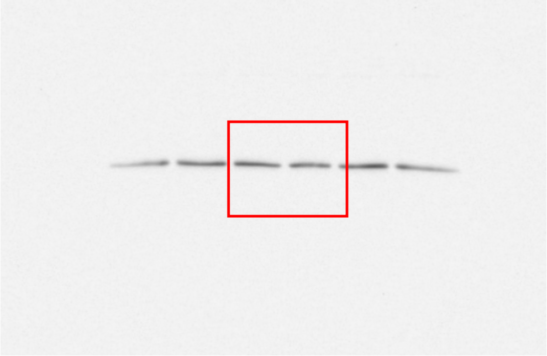 |
